# Molecular characterization of MHC class IIB genes of sympatric Neotropical cichlids

**DOI:** 10.1186/s12863-017-0474-x

**Published:** 2017-02-15

**Authors:** Melinda J. Hofmann, Seraina E. Bracamonte, Christophe Eizaguirre, Marta Barluenga

**Affiliations:** 1 Museo Nacional de Ciencias Naturales, CSIC, José Gutiérrez Abascal, 2, 28006 Madrid, Spain; 2 GEOMAR Helmholtz Centre for Ocean Research Kiel, Duesternbrooker weg 20, 24105 Kiel, Germany; 3 Leibniz-Institute of Freshwater Ecology and Inland Fisheries, Müggelseedamm 310, 12587 Berlin, Germany; 4 Queen Mary University of London, School of Biological and Chemical Sciences, Mile End Road, London, E1 4NS UK

**Keywords:** Major Histocompatibility Complex, Sympatric, Neotropical, Midas cichlid fish, *Amphilophus*

## Abstract

**Background:**

The Major Histocompatibility Complex (MHC) is a key component of the adaptive immune system of all vertebrates and consists of the most polymorphic genes known to date. Due to this complexity, however, MHC remains to be characterized in many species including any Neotropical cichlid fish. Neotropical crater lake cichlids are ideal models to study evolutionary processes as they display one of the most convincing examples of sympatric and repeated parallel radiation events within and among isolated crater lakes.

**Results:**

Here, we characterized the genes of MHC class IIB chain of the Midas cichlid species complex (*Amphilophus* cf. *citrinellus)* including fish from five lakes in Nicaragua*.* We designed 19 new specific primers anchored in a stepwise fashion in order to detect all alleles present. We obtained 866 genomic DNA (gDNA) sequences from thirteen individuals and 756 additional sequences from complementary DNA (cDNA) of seven of those individuals. We identified 69 distinct alleles with up to 25 alleles per individual. We also found considerable intron length variation and mismatches of alleles detected in cDNA and gDNA suggesting that some loci have undergone pseudogenization. Lastly, we created a model of protein structure homology for each allele and identified their key structural components.

**Conclusions:**

Overall, the Midas cichlid has one of the most diverse repertoires of MHC class IIB genes known, which could serve as a powerful tool to elucidate the process of divergent radiations, colonization and speciation in sympatry.

**Electronic supplementary material:**

The online version of this article (doi:10.1186/s12863-017-0474-x) contains supplementary material, which is available to authorized users.

## Background

The Major Histocompatibility Complex (MHC) is a key component of the adaptive immune system of all jawed vertebrates [1, 2]. The function of the MHC molecules is to present short self and non-self peptides often derived from parasites and pathogens for recognition by T-lymphocytes [3]. This sets off the cascade of targeted immune defenses against those specific parasites and pathogens. MHC also plays a role in establishing a memory to rapidly eliminate those agents in case of future encounters [3]. MHC molecules are encoded by the most polymorphic genes in all jawed vertebrates, and most species have different number of loci that are co-dominantly expressed (e.g. [4, 5]). There are two classical antigen presenting MHC molecules, MHC class I, that is expressed on all nucleated cells and elicits a response against intracellular parasites, and MHC class II, that is only expressed on antigen-presenting cells (macrophages, B-cells and dendritic cells), which actively engulf and process inter-cellular parasites [3]. Here, we focus on MHC class II which is composed of two chains, α and β, which together form the peptide-binding groove [4]. The peptide-binding region of the β chain is the most polymorphic and hence the most studied region of the MHC. In general, the MHC IIB region is divided into 5 to 6 exons with varying intron lengths depending on species and haplotypes [5–7].

The highly polymorphic multigene nature of MHC causes some technical difficulties when trying to simultaneously detect all alleles, particularly those that are rare in the target population. Cloning and Sanger sequencing have associated PCR-based errors and PCR amplification biases [8–10], making accurate amplification a laborious and costly process. Next generation sequencing technologies have, to some extent, facilitated population level studies of MHC, although those new techniques tend to overestimate allelic diversity [11, 12]. Overcoming those challenges allows the use of MHC as a powerful tool to study biodiversity [13, 14], disease dynamics [15], evolutionary processes [16, 17], and even to estimate the number of founders of a population [18].

Cichlid fish are excellent model systems to study evolutionary processes since they demonstrate some of the most extreme examples of explosive adaptive radiations (e.g. [19–22]). They are some of the most species-rich families of freshwater fishes worldwide, and their hotspots of diversification are the great lakes of East Africa. They are also present in Central and South America [23, 24]. Particularly, the Neotropical Midas cichlid species complex (*Amphilophus spp.*) is a valuable model system for the study of recent speciation [25–27]. This group not only comprises one of the most compelling examples of sympatric speciation [28], but also recent independent colonization events and in situ rapid diversification [29], which makes it an excellent natural experiment of adaptation and incipient speciation [25].

Many studies have attributed cichlid’s rapid speciation events to various factors, including phenotypic plasticity [30], reproductive behavior and local adaptation [31, 32], and even genomic processes [33–35]. It has been suggested that the mechanism of adaptive speciation in general, and in sympatry in particular, may result from a pleiotropic role of the MHC in co-evolutionary dynamics of local host-parasites and odor-mediated mate choice ultimately leading to reproductive isolation [14, 36, 37]. Here, we characterized the β chain of the MHC class II in the Neotropical Midas cichlid species complex to establish the baseline for evaluating the role of parasites and immune system in sympatric speciation.

A striking characteristic of MHC polymorphism is the occurrence of similar alleles in related species, known as trans-species polymorphism (TSP) [38]. This similarity might have arisen by convergence [39, 40], although a more commonly accepted idea is that this polymorphism is maintained, mostly by balancing selection, beyond the species formation [41]. This polymorphism transmitted through several speciation phases can be a useful tool to study speciation itself [41]. TSP has been found to occur across related species of reptiles [42], mammals [43], amphibians [44], birds [45, 46], and fish [47]. In this study we also characterize events of TSP.

There is some knowledge about MHC diversity patterns of cichlid fish [37, 48–51], but this comes exclusively from African species. Some studies have focused on the diversity of MHC class I in cichlids from Lake Victoria, finding many common alleles across species [52]. Other studies have found high diversity of MHC class IIB alleles in different species of Lake Malawi cichlids [48, 53]. A population genetic analysis on MHC class IIB of Lake Malawi cichlids even suggested that adaptive divergence at this locus could be linked to speciation in cichlids [36].

Old and New World cichlids have been geographically separated for a very long time [54–56], therefore MHC evolution in Neotropical cichlids is likely to have followed its own evolutionary trajectory. Therefore, MHC has to be characterized *de novo* in the Midas cichlid in order to understand its role in their adaptation and speciation. We first sequenced exons 1–6 of the MHC IIB and described intron and exon conformation as well as most intron length variability. Then we used both genomic (gDNA) and expressed transcripts (mRNA) to characterize the allelic diversity existing in exon 2 – that which encodes for the peptide binding groove. We then tested for various modes of evolution of the MHC and modeled the tertiary structure of each detected allele to identify the structural components of the MHC molecules.

## Methods

### Sampling, DNA and RNA isolation

**Fig. 1:**
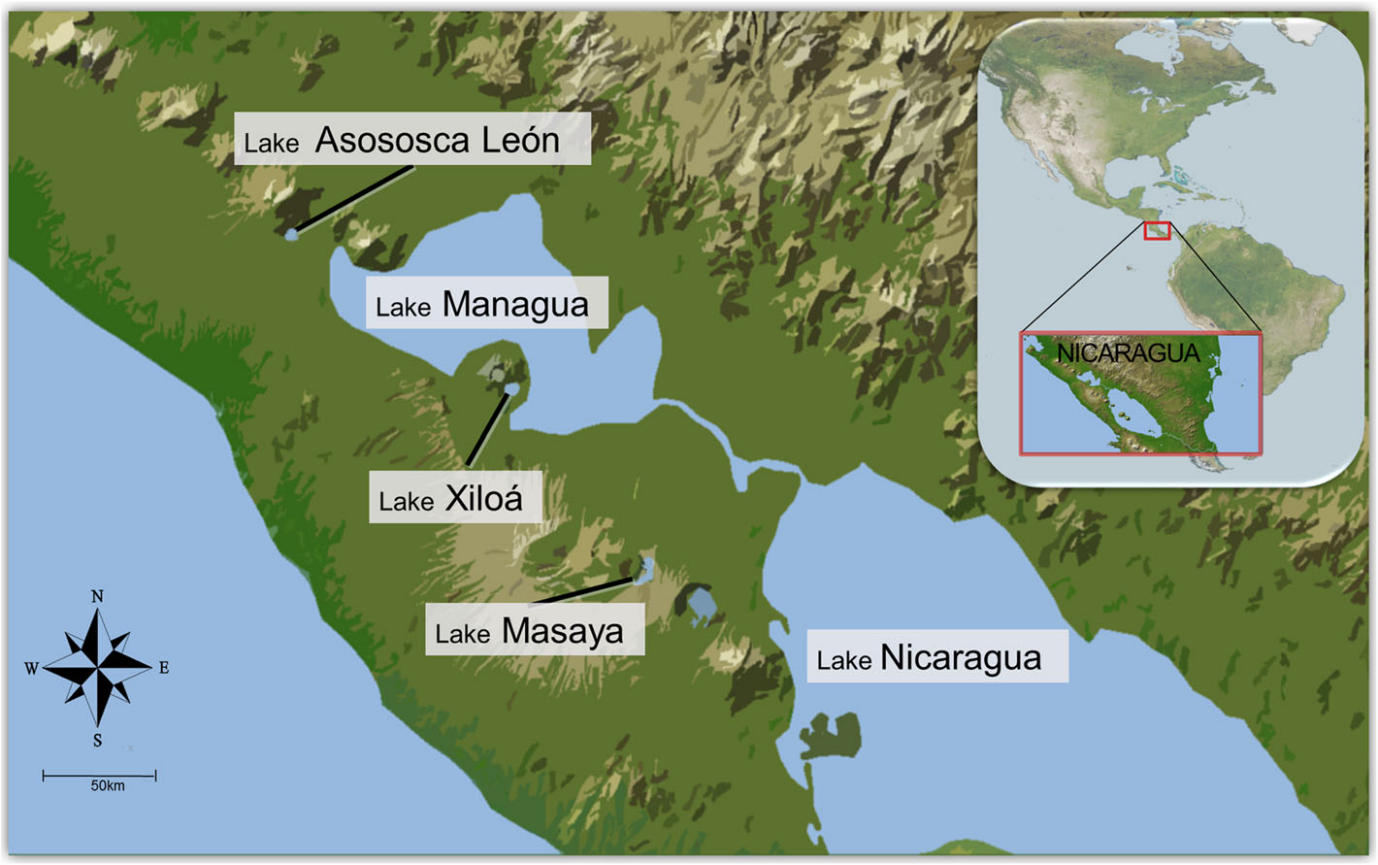
Map of the Pacific coast of Nicaragua (Central America) with the great lakes and several crater lakes where samples were collected (source http://photojournal.jpl.nasa.gov/catalog/PIA03364; wikimedia.org). Samples belonged to the four species *Amphilophus citrinellus, A. labiatus, A. amarillo* and *A. xiloaensis*

Sampling of Midas cichlid fish took place in several Nicaraguan lakes (Fig. 1). Adult fish were captured using gill nets (collection permit number 001-012012), anesthetized with MS 222 following standard procedures and euthanized on ice before processing. Fin tissues of 13 randomly selected individuals were preserved in 100% ethanol at 4 °C. Those 13 samples represent a good portion of the diversity of this species complex (Fig. 1, Additional file 1: Table S1). Additional spleen tissue samples of 7 of these individuals was preserved in RNAlater® (Qiagen, Hilden, Germany) and stored at -80 °C.

Total genomic DNA (gDNA) was extracted using DNeasy spin columns for Blood and Tissue Kit® (Qiagen, Hilden, Germany) according to the manufacturer’s protocol, with the addition of RNAse. DNA was quantified using Nanodrop 1000 (ThermoFisher Scientific, Bonn) and standardized to a concentration of 20 ng / μl. RNA was extracted with Invitrap Spin tissue RNA mini kit® (Berlin, Germany) and the reverse transcription performed with the QuantiTect® Reverse Transcription kit (Qiagen, Hilden, Germany).

### Primers design

Firstly, to characterize the allelic diversity in the exon 2 of the MHC IIB gene in the Midas cichlid, we retrieved MHC sequences from GenBank choosing different sequences from related fish species with well-characterized MHC genes. We aligned the sequences (see Additional file 1: Table S2) and designed a reverse primer (MHC_Rev3 “GATCTGTTTGGGGTAGAAGTCG”) located in the most conserved region in the middle of exon 3. We did a PCR by pairing this reverse primer with two published forward primers designed for sticklebacks located in conserved upstream regions of exon 2 (SatoGa11_mod1 [57], GAIIEx2startF [58]). Using the resulting sequences we designed 14 additional primers in a stepwise manner. We designed new primers in adjacent regions of the sequence, considered sets of new amplicons, and designed additional primers on the new sequences maximizing allele amplification (Table 1). We paired all forward and reverse primers. Additionally, we specifically designed 4 primers (AcMHCIIF3, AcMHCIIF4, AcMHCIIF5, and AcMHCIIF6) to discriminate a group of particularly abundant alleles that were not very variable (later referred to as Group I), which could hinder the detection of rarer alleles.

**Table 1:** Primer combinations used for amplification of MHC IIB

Forward	Primer Reverse Primer	TA	Fragment
SatoGa11_mod1	MHC Rev3	59 °C	E2 - E3
AACTCCACKGAKCTGAAGRAC	GATCTGTTTGGGGTAGAAGTCG		
GAIIEx2startF	MHC Rev3	55 °C	E2 - E3
GTCTTTAACTCCACGGAGCTGAAGG	GATCTGTTTGGGGTAGAAGTCG		
GAIIEx2startF	AcMHCIIBR1	59 °C	E2 - E3
GTCTTTAACTCCACGGAGCTGAAGG	GGRGTGAAGTCTGACTRATGG		
AaMHCIIBE1F1b	MHC Rev3	56 °C	E1 - E3
ATGGCTYCATCCTTYMTC	GATCTGTTTGGGGTAGAAGTCG		
AaMHCIIBE1F1b	AcMHCIIBR4	59 °C	E1 - E5
ATGGCTYCATCCTTYMTC	ACCCAGGATCAGTCCTGAGG		
AcMHCIIBF2 (excludes group I)	AcMHCIIBR3 (excludes gop I)	64 °C	E2 - E2/I2
TTAACTCCACKGAGCTGAASGACA	GAYGATGAAYCATAACTCACCTGAT		
AcMHCIIBF3 (only group I)	AcMHCIIBR4	46 °C	E2/I2 - E5
TCAGGTGAGTYATGDTTCATC	ACCCAGGATCAGTCCTGAGG		
AcMHCIIBF3 (only group I)	AcMHCIIBR5	44 °C	E2/I2 - R5
TCAGGTGAGTYATGDTTCATC	TTCCTCTTGTAGTAGATGAATCC		
AcMHCIIBF4 (excludes goup I)	AcMHCIIBR4	54 °C	E2/I2 - E5
TCAGGTGAGTCTGTTTCTGTG	ACCCAGGATCAGTCCTGAGG		
AcMHCIIBF5 (excludes group I)	MHC Rev3	55 °C	E2 - E3
CCACKGAGCTGAASGACATSGAG	GATCTGTTTGGGGTAGAAGTCG		
AcMHCIIBF5 (excludes group I)	AcMHCIIBR4	59 °C	E2 - E5
CCACKGAGCTGAASGACATSGAG	ACCCAGGATCAGTCCTGAGG		
AcMHCIIBF7	AcMHCIIBR9	50 °C	E2 - E2
CGAGTACGTTCGATCTTTGTATTGC	DCTGATTTAGTCAGAGCAGTCT		
AcMHCIIBF8	AcMHCIIBR9	55 °C	E2 - E2
CGAGTWCATCARVTCTTACTAYTWC	DCTGATTTAGTCAGAGCAGTCT		
AcMHCIIBF9	AcMHCIIBR8	NA	I2 - I2
GAAACCTGTTCACAGCAGTCCCTC	CATGTGCTACATGCAACATATCA		
AcMHCIIBF6	MHC Rev3	55 °C	E2 - E3
CCACTGAGCTGAASGACATSGAG	GATCTGTTTGGGGTAGAAGTCG		

*Fragment* indicates the region of the gene amplified: ‘E’ exon, ‘I’ intron, ‘/’ exon-intron boundary. The primers AcMHCIIBF9 and AcMHCIIBR8 were used only to sequence through clones with long intron 2

### PCR Amplification and gel extraction

PCR amplifications were performed following the recommendations of Lenz and Becker [10] in order to reduce PCR artifacts, common in the amplification of multigene families. We used Taq Polymerase with no proof-reading capacity, extended elongation times, excess of primers to avoid incomplete amplicons acting as heteroduplex primers, and duplicated reactions. However, we did not reduce PCR cycles or do a reconditioning PCR since under those recommendations our bands were too weak for cloning. Each amplification was done in two independent reactions, each consisting of 2 μl 10x Dreamtaq Buffer, 1 μl dNTP’s (10 mM), 2 μl of each primer (5 pmol / μl), 0.2 μl Taq Polymerase (Dreamtaq®) and 2 μl of template DNA in a total volume of 20 μl. The thermal profile started with an initial denaturation step of 95 °C for 3 min, followed by 30 cycles of denaturation at 94 °C for 30 s, annealing at specific temperature for each primer pair (ranging from 44 to 64 °C, Table 1) for 1 min, elongation at 72 °C for 1 min, with a final elongation at 72 °C for 10 min. The PCR reactions for each individual and primer pair were then pooled and loaded into a 2% agarose gel and run at 40 V for 4 h. Gels were then stained with Ethidium bromide to visualize bands. The bands of interest were cut and extracted with NucleoSpin Gel and PCR Clean-up® (MACHEREY-NAGEL GmbH & Co. KG) for further cloning.

### Cloning

PCR amplicons were cloned with the Qiagen PCR cloning Kit® (Qiagen, Hilden, Germany). Cloning followed the protocol described in Bracamonte et al. [59]. For clone screening, 1 μl of the denatured clones was used as template for a PCR with the universal M13 primers: M13_Funi (5′ACGACGTTGTAAAACGACGGCCAG 3′), and M13_RP15 (5′TTCACACAGGAAACAGCTATGACC 3′). The reaction had a final volume of 10 μl, included 1 μl 10x Dreamtaq Buffer, 0.5 μl dNTPs (10 mM), 1 μl of each primer (5 pmol / μl), 0.1 μl Taq Polymerase (Dreamtaq®), and ran using the following thermal profile: initial denaturing step at 95 °C for 1 min, followed by 25 cycles of denaturing at 96 °C for 10 s, annealing at 50 °C for 10 s, elongation at 72 °C for 1 min, with a final elongation at 72 °C for 7 min. Two μl of this PCR product were then loaded in a 1% agarose gel and run for 30 min at 90 V, and ultimately stained with Ethidium bromide to visualize bands. We sequenced the clones that were positive for the amplicon. We sequenced between 16 and 48 clones per amplification in order to detect rare alleles.

### Sequencing

Cycle sequencing was done using the Big Dye Terminator v3.1 using the Cycle Sequencing Kit (Life Technologies) following the manufacturer’s protocol scaled to 10 μl total reaction volume per sample. The product was then purified using 50 μl BigDye X Terminator^TM^ Purification Kit® mix (Life technologies, Thermo Fisher Scientific Inc). Sequencing took place on an ABI 3730 Genetic Analyzer (Life Technologies). Even though MHC sequence variants may stem from different loci and therefore may be paralogs, we will refer to them as alleles.

### Identifying and naming alleles

The taxonomic status of the species within the Midas cichlid complex is under considerable debate. Although some species have been described recently within this species complex, due to their very recent history (<50000 years) we name our alleles under the generic name “Amci” for *Amphilophus* cf*. citrinellus*. Sequences were aligned using CODON-CODE ALIGNER® (Codoncode Corporation 2002–2009) and analyzed with BIOEDIT v1 [60]. To avoid sequence artefacts, generally alleles were only considered true alleles if they were amplified in at least two independent PCR and cloning events (see Results for exceptions on this). We manually inspected the alignment and removed all sequences that were identified as chimeras by being partially identical to one allele group, and partially identical to another allele group and only existing in one PCR product. Sequences identified as chimeras were removed from all further analyses. A consensus was created from all identical sequences of different lengths and this was hereafter considered an allele. All alleles were aligned with well annotated published sequences [58] and checked for stop codons within exons to further rule out the presence of pseudogenes. Since there is no complete genome for this species, identifying the family and locus that each allele belonged to is not currently possible. Alleles were named following the allele nomenclature guidelines for MHC established by Klein et al. [61]. We looked for tandem repeats in all alleles using Tandem Repeat Finder v4.09 [62].

We did a blast search of all alleles on the *Amphilophus citrinellus* draft genome shotgun sequencing project (BioProject PRJEB6974) [63] to confirm the exon structure of the MHC IIB alleles we found and to confirm intron length variability.

Alleles were assigned to groups according to estimates of evolutionary divergence between sequences. Analyses were conducted using the Maximum Composite Likelihood model [64] in MEGA v5.2 [65]. Codon positions included were 1st, 2nd, 3rd, and noncoding. All positions containing gaps and missing data were eliminated. The final dataset had a total of 201 positions. We validated the resulting groups with a randomization analysis in R [66]. The mean pairwise distances within each group was compared to a null distribution generated by a random selection of equivalent alleles repeated 999 times to calculate the p-value. The results of this analysis were contrasted with the phylogenetic reconstruction of the allele relationships.

### Phylogenetic and statistical analyses

In order to determine the phylogenetic relationships between alleles, we used exon 2 and 3 to construct a phylogenetic tree using Bayesian inference with BEAST v2.0 [67]. We found the most appropriate substitution model (HKY + G) and partitioning scheme according to the Bayesian Information Criterion implemented in PARTITIONFINDER [68]. We specified the parameters in BEAUti v2.0 [67] for the BEAST-run, and the MCMCs were run for 10^9^ generations sampling every 100,000 trees. We used a strict clock model and a Yule speciation prior. We inspected the traces for convergence with TRACER 1.5 (http://tree.bio.ed.ac.uk/software/tracer/) and checked that they were higher than 200. The 10,000 resulting trees were summarized with TREEANNOTATOR v2.1.2 (http://beast.bio.ed.ac.uk/treeannotator) applying a 10% burn-in. We depicted the phylogeny with the corresponding posterior values of each node with FIGTREE v1.4.2 (http://tree.bio.ed.ac.uk/software/figtree/).

To further analyze the relationship between the alleles we constructed a neighbor-net network with SPLITSTREE4 [69]. We calibrated the network by calculating the best fit model using MEGA and estimated the probability of invariable sites.

To evaluate TSP we gathered previously published sequences from several fish species. We selected 11 sequences of MHC IIB of a well-studied African cichlid, Nile tilapia (*Oreochromis niloticus*), from Sato et al. [70], and 9 sequences from a search at NCBI’s Genbank database with BLASTx from other cichlids and two other fish families: Sciaenidae and Sebastidae which had alleles closest to cichlid ones (Additional file 1: Table S3). We constructed a phylogeny with all the alleles (69 from the Midas cichlid and 20 from other species) using the same methodology and parameters as described above.

### Tests of selection

In order to elucidate on the evolutionary history of MHC IIB of Midas cichlid, to determine if different selective pressures have shaped the different domains of the molecule, and if selection has acted differently on the different groups of alleles, we performed selection tests, both by domain and on the entire sequence, as implemented in MEGA. We acknowledge the limitations of these tests given the fact that we cannot allocate alleles to specific loci. Selection tests were performed on the groups of alleles established by sequence divergence. We tested separately the alleles of Group I since they appear to follow a different evolutionary pathway. We calculated rates of substitution (d_N_ and d_S_), and tested for overall positive or purifying selection with a Z-test of selection for each domain separately. For alleles in Group I we applied the Pamilo-Bianchi-Li method with Kimura 2-parameter correction, and for the rest of alleles we applied the Nei-Gojobori method with Jukes-Cantor correction, in accordance with their corresponding best fit substitution model. Significance levels were estimated with 10,000 bootstrap replicates. We used the Nei-Gojobori method for calculating the “absolute” number of synonymous and non-synonymous sites since the Pamilo-Bianchi-Li method is not available for this test. We do not have the full sequence coverage for all alleles, and therefore we used a pairwise method that compares two sequences at a time and then averages over all possible comparisons. With this method we could compare all 69 allele despite some not having complete sequences.

To further evaluate the mode of evolution of each group of alleles, to identify possible peptide binding sites, and to identify potentially non-classical MHC alleles, we used site-specific tests of selection focusing on exons 2 and 3 that are the exons for which we have full sequence coverage for most alleles. We tested site-specific positive selection within each of the four groups of alleles, and across all alleles excluding Group I, due to its likely different evolutionary history. Site-specific selection was tested using CodeML in PAML v4.7 [71], assuming different ω parameters among codons with no a priori knowledge of which class of selection (neutral, purifying, or positive) a given codon belongs to. We estimated parameters under five different codon substitution models: Beta models M7 (no positive selection), M8 (positive selection), and M8a (no positive selection and ω = 1), and models M1a (nearly neutral) and M2a (positive selection) [71]. A likelihood ratio test (LRT) was performed to compare the fit of the models with and without selection. Statistical significance was determined by comparing twice the difference of log-likelihood scores (2ΔlnL) to the *Χ* ^2^ distribution with degrees equal to the difference in the number of parameters between the models to be compared.

### Protein tertiary structure homology models

To characterize the tertiary structure of each allele, and determine if they have the proper characteristics to allow them to fold into a potentially functional MHC molecule we built protein homology models using the Swiss-Model workspace v8.05 [72]. This method identifies structural templates from a protein data bank, aligns the target sequence to a template structure, builds a 3D model, and evaluates the quality of the model. The parameters considered were: *overall model quality* (QMEAN4) in which less negative values indicate more reliable homology models, and *global model quality estimation* (GMQE) in which the higher numbers indicate the more reliable models. We also located the four conserved cysteine residues that are essential for the stability of the β_1_ and β_2_ domains of MHC IIB, and analyzed the structure of the N- and C-terminal areas of the protein.

## Results

### Cloning, sequencing and MHC organization

**Fig. 2:**
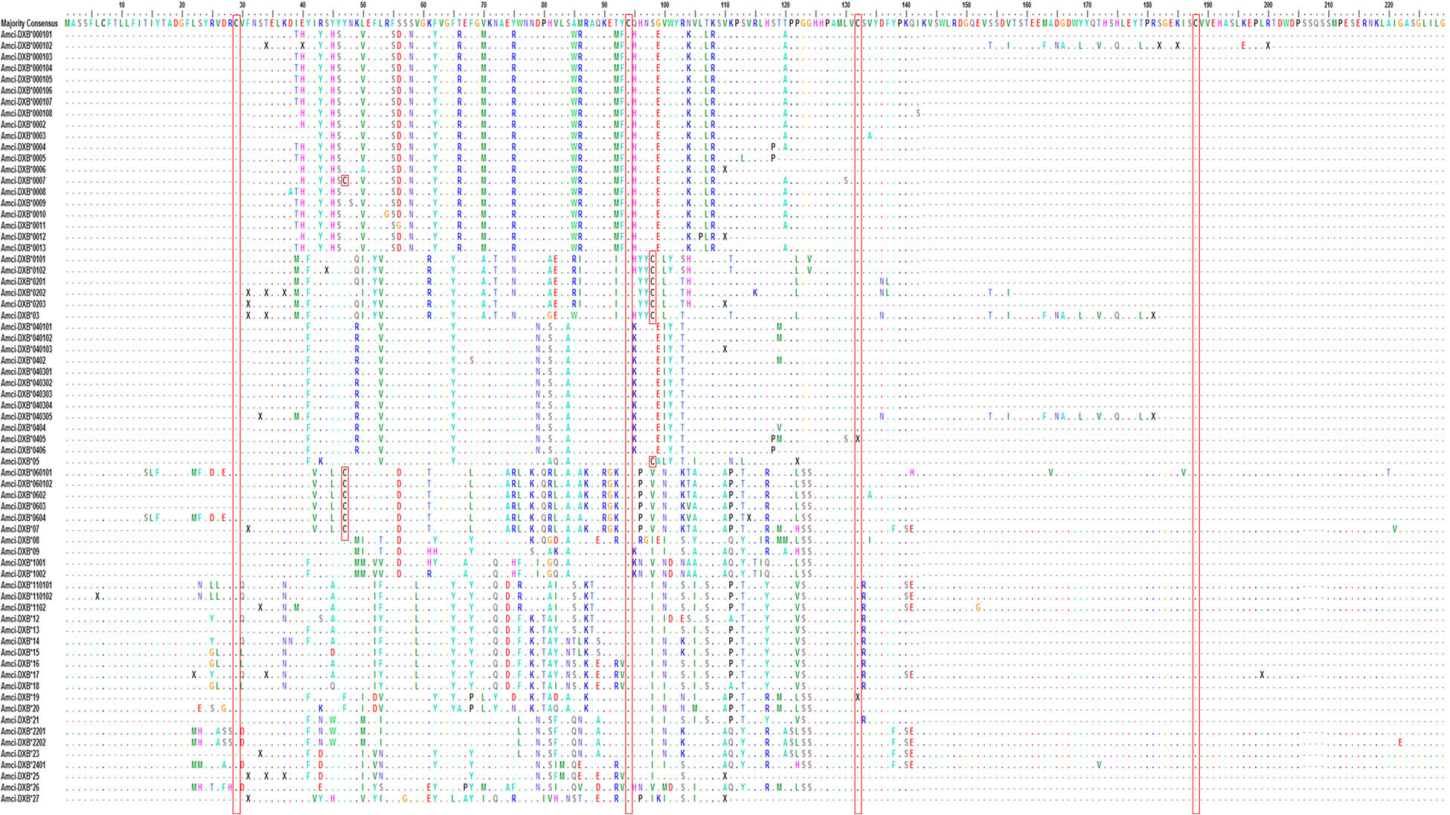
Alignment of amino acid sequences of MHC IIB. A majority consensus sequence was made for comparison. ‘•’ represent identical amino acids, ‘-’ represent gaps or introns that were not sequenced. Cysteine residues are outlined in red boxes

**Fig. 3:**
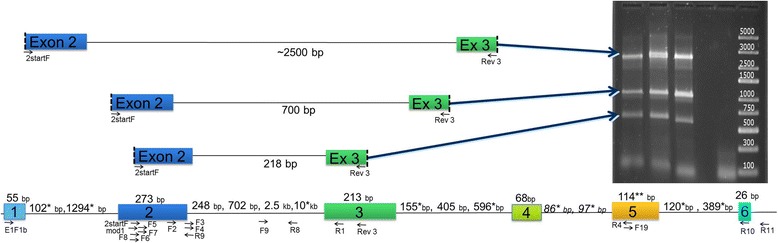
Representation of intron-exon organization of MHC IIB sequences showing length of fragments and position of primers. The gel shows variation in the length of intron 2 (showing exon 2 / intron 2 / exon 3) for three individuals. * indicates lengths observed in sequences of the *Amphilophus citrinellus* draft genome shotgun sequencing project and ** indicate the exon that we found to be 12 bp longer in allele DXB*060101 and DXB*07

For a total of 13 individuals we obtained 867 gDNA and 756 cDNA sequences, all belonging to the MHC IIB. These sequences revealed 69 distinct alleles (GenBank accession numbers: KY039442-KY039474 and KY354964-KY355011). Three of these alleles (DXB*05, DXB*1002 and DXB*13) were only represented by one single sequence while one was only represented by 2 sequences (DXB*0402), and would remain to be confirmed by an additional PCR amplification. We found an average of 12.5 (SD = 6.1) alleles per individual and a maximum of 25 (Table 2 and Fig. 2). We identified six exons and five introns, but we did not sequence all exons for all alleles (Additional file 1: Table S4). We characterized the gene from exon 1 through exon 6 focusing efforts on exon 2 where we expect most variability (Fig. 2). For fifteen alleles we amplified exon 1 and found that it has 55 bp, and includes the start codon “ATG” that codes for a methionine residue at the N-terminus. Exon 1 mainly codes for the signal peptide formed by 16–20 amino acids. Exon 2 is the most polymorphic of all exons with up to ten amino acids per site (Additional file 1: Figure S1), and consists of 273 bp, of which we sequenced at least 231 bp for all 69 alleles. Exon 2 encodes the beta sheets and alpha helixes of the β_1_ domain of the mature MHC class II molecule. For most alleles we amplified intron 2 which revealed extreme length variation ranging from 200 to 2500 bp (Fig. 3). Alleles with long intron 2 contained three tandem repeats, a 42mer repeated 6.4 times, a 21mer repeated 12.6–18.6 times, and a 127mer repeated twice which contributed to their length. For exon 3 we amplified from 99 bp to 214 bp of most alleles, exon 3 is 214 bp long and is enriched in the amino acid proline, aiding in the formation of beta turns that are essential to the structure of the β_2_ domain. We obtained partial sequences of intron 3, for two alleles, and a complete sequence for one allele that was 405 bp long. We obtained sequences of exon 4 for ten alleles that were 69 bp. For nine alleles we sequenced 64 bp of exon 5 which is expected to have 114 bp. Two of them (DXB*060101 and DXB^*^07) have additional 12 bp on the 5′end of exon 5. Introns 4 and 5 were not sequenced for any allele because of their known low variability [2]. We generated only partial sequences of exon 6, which we cannot assign to individual alleles as they do not span any allele specific polymorphisms due to the low variability of this region. The summary of the length in bp of each sequenced intron and exon in shown in Additional file 1: Table S4 with the reference of the sequence of *Oreochromis niloticus*.

**Table 2:** List of all of MHC IIB alleles found in the Midas cichlid

Samples	AM05	AM06	AH65	AL11	AL64	AQ72	27D3		25D8		27G4	25A2		25D1		25B2		27E7		Total Sequences
Template Allele	gDNA	gDNA	gDNA	gDNA	gDNA	gDNA	gDNA	cDNA	gDNA	cDNA	cDNA	gDNA	cDNA	gDNA	cDNA	gDNA	cDNA	gDNA	cDNA	
Amci-DXB*000101	12	13	69	3		21	46													164
Amci-DXB*000102	71	10	49		13	77	4													224
Amci-DXB*000103	1					1														2
Amci-DXB*000104	1			1																2
Amci-DXB*000105	1		1																	2
Amci-DXB*000106		1				2														3
Amci-DXB*000107			1			1														2
Amci-DXB*000108			1			1														2
Amci-DXB*0002	1				1															2
Amci-DXB*0003	1		1									1								3
Amci-DXB*0004			1	1		1	13													16
Amci-DXB*0005							2													2
Amci-DXB*0006			2			1	2													5
Amci-DXB*0007	1				1															2
Amci-DXB*0008	2	1			1															4
Amci-DXB*0009			1			1														2
Amci-DXB*0010				1		1														2
Amci-DXB*0011		1	1																	2
Amci-DXB*0012			1			1	1													3
Amci-DXB*0013			1	1																2
Amci-DXB*0101			2	2		3	1		2											10
Amci-DXB*0102	5		2	2	1	9														19
Amci-DXB*0201							39					1				4				44
Amci-DXB*0202							17					14				26	1			58
Amci-DXB*0203									6			6								12
Amci-DXB*03												26				1				27
Amci-DXB*040101	9	1	4		2	13	7		4			9				10		2		61
Amci-DXB*040102	5	1	5	3	1						17				3		8			43
Amci-DXB*040103	1					1														2
Amci-DXB*0402	1					1														2
Amci-DXB*040301				3	2	1		1		25					3		7			42
Amci-DXB*040302			1			1														2
Amci-DXB*040303	2					16	13													31
Amci-DXB*040304				3	8		18													29
Amci-DXB*040305												4								4
Amci-DXB*0404	2					1														3
Amci-DXB*0405							3	26				1			16		20			66
Amci-DXB*0406								1							1					2
Amci-DXB*05			1																	1
Amci-DXB*060101								1					43	1	31	2	42		16	136
Amci-DXB*060102													1		1					2
Amci-DXB*0602													1		6					7
Amci-DXB*0603										5									3	8
Amci-DXB*0604		1													1				16	18
Amci-DXB*07													2							2
Amci-DXB*08														1	40					41
Amci-DXB*09										50									9	59
Amci-DXB*1001															1				25	26
Amci-DXB*1002															1					1
Amci-DXB*110101							31	42												73
Amci-DXB*110102								2												2
Amci-DXB*1102								8												8
Amci-DXB*12										1					19					20
Amci-DXB*13											1									1
Amci-DXB*14																			38	38
Amci-DXB*15										16										16
Amci-DXB*16							4								34					38
Amci-DXB*17							4	18												22
Amci-DXB*18												17	44			23	50			134
Amci-DXB*19																	9			9
Amci-DXB*20																			2	2
Amci-DXB*21															2					2
Amci-DXB*2201							2	23												25
Amci-DXB*2202								2												2
Amci-DXB*23								2												2
Amci-DXB*2401								13												13
Amci-DXB*25												1				3				4
Amci-DXB*26										6										6
Amci-DXB*27	2																			2
Total sequences	118	29	144	20	30	154	207	139	12	103	18	80	91	2	159	69	137	2	109	1623
Total Alleles	17	8	18	10	9	20	25		9		2	14		14		11		8		69

Alleles found in 13 Midas cichlid samples (listed on the top) showing number of copies recovered per allele amplified in gDNA and cDNA in each individual. All alleles except DXB*05, DXB*1002, DXB*0402, and DXB*13 were confirmed with at least two independent PCR reactions. At the bottom we summarize total number of sequences and total number of alleles obtained

To further corroborate our results, we did a BLAST search on the *A. citrinellus* shotgun genome project (BioProject PRJEB6974) and recovered three complete MHC IIB genes from contig 1595 position 54549-67334 (CCOE01001596.1), contig 1079 position 91043-98760 (CCOE01001080.1), and unplaced scaffold 125 position 28791-32030 (CCOE01001892.1), as well as several incomplete MHC IIB sequences. The three complete sequences contained all 6 exons, with the same length we describe. This data confirms that the length of exon 5 is 114 bp (of which we only retrieved partial sequences), but only contained one of the two variants we found (the short variant). Intron length variability was also represented in the genome sequences. We did not find in this data all intron variants we found in our sequences (e.g. we found intron 2 with 702 bp and 1.5 kb, but not with 248 bp), but found new intron length variants (e.g. intron 2 of contig 1595 is 10 kb). This data must be taken with caution since the shotgun sequences have not been confirmed with PCR and sequencing standard protocols employed when working with MHC. Nevertheless, this data provides valuable corroboration about intron lengths that is otherwise unavailable at this time.

Although in general alleles are defined as alternate sequences of a single locus, since there is no complete reference genome for this species, there is no information on how many MHC loci to expect, or how variable they may be. It must be noted that our alleles cannot be assigned to specific loci at this stage.

### Grouping of alleles and Phylogenetic analyses

**Fig. 4:**
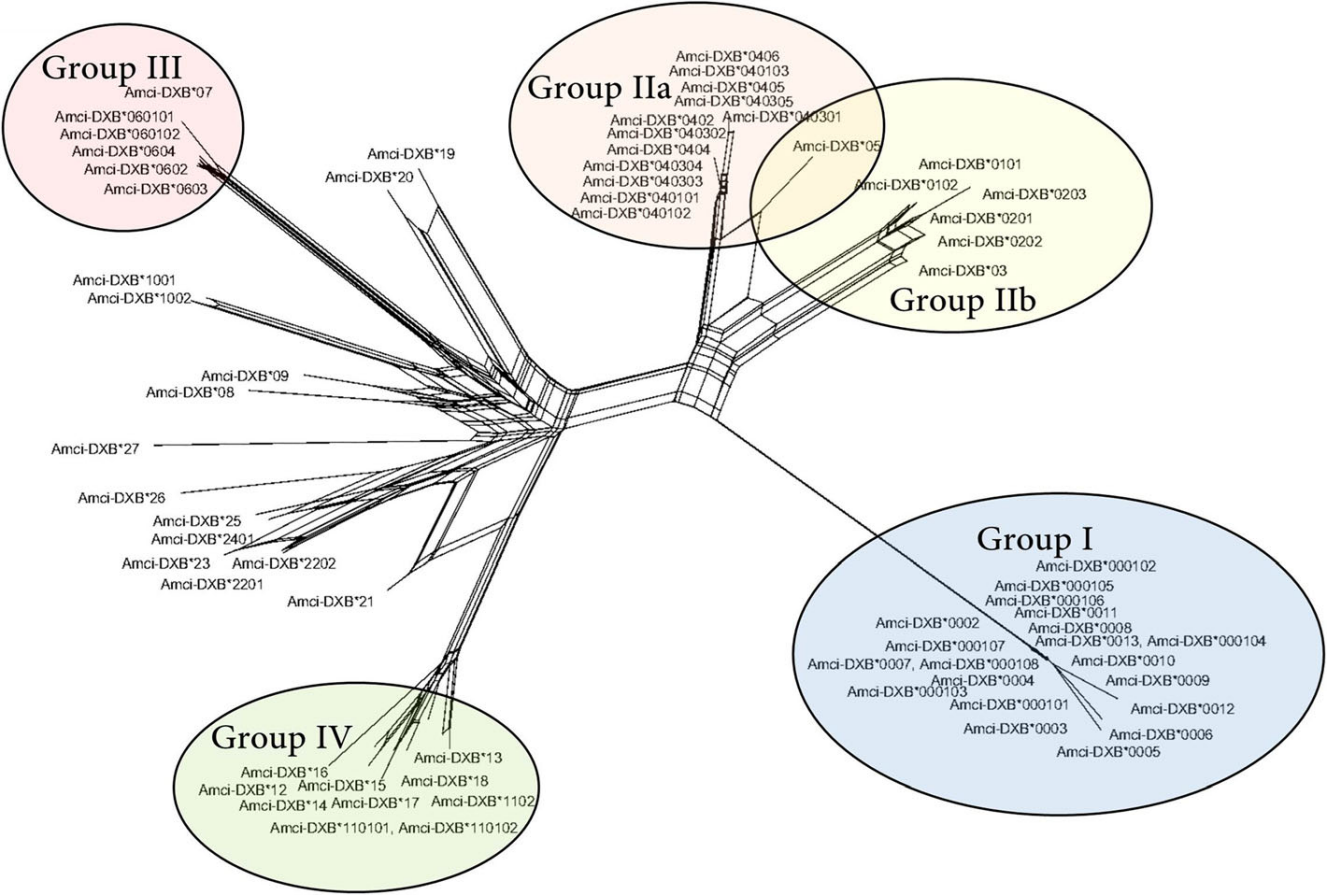
Neighbor-net network based on exons 2 and 3 of MHC IIB allele relationships. Groups of alleles found with estimates of evolutionary divergence between sequences are shown

We constructed a phylogeny with all 69 alleles recovered from exons 2 and 3 (Additional file 1: Figure S2). We also constructed a neighbor-net network (Fig. 4). According to estimates of evolutionary divergence between sequences, alleles grouped into five major groups (Additional file 1: Figure S3). When plotting these groups into the network one of these groups was not consistent and we left them as ungrouped (14 alleles). The remaining alleles clustered in four groups (I, II, III, IV). The results of the randomization analysis validating the groups are shown in Additional file 1: Table S5. Group I grouped 20 alleles, which were only found in gDNA. These alleles showed very low polymorphism and might represent non-classical MHC genes or MHC pseudogenes (see selection analyses section). Group II was further divided into two sub-groups, II-a and II-b. Group II-a assembled 13 alleles, 9 found in gDNA, 1 found in cDNA, and 3 found in both cDNA and gDNA. Group II-b had 6 alleles, 5 found in gDNA and 1 found in both gDNA and cDNA. Group III assembled 6 alleles, 5 found in cDNA and 1 found in both gDNA and cDNA. Ten alleles were included in Group IV, 7 found in cDNA, and 3 in both gDNA and cDNA. From the 14 ungrouped alleles, 12 were found in cDNA, 1 in gDNA, and 1 in both cDNA and gDNA.

Taken together the Midas cichlid MHC IIB alleles belonged to two types, those alleles not found expressed in the tissue examined in this study (alleles in Group I), and those found in both gDNA and cDNA (alleles in the rest of the groups).

### Trans-species polymorphism

**Fig. 5:**
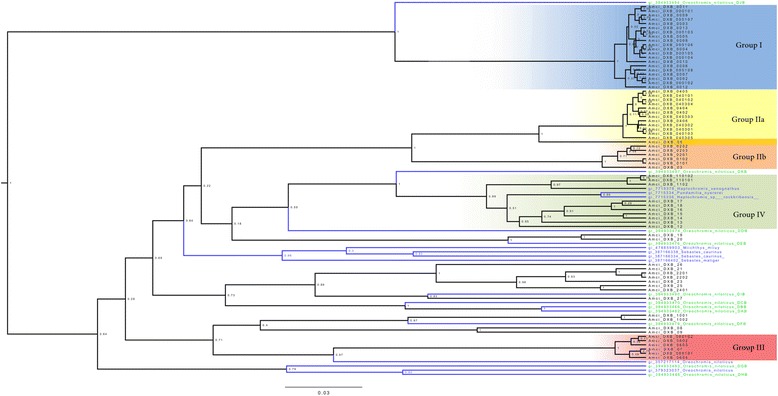
Phylogenetic tree showing trans-species polymorphisim of exons 2 and 3 of all MHC IIB alleles found in the Midas cichlid (in black) and 20 alleles from other species (in blue), with posterior probabilities. Alleles are grouped according to how they cluster in Fig. 4

We reconstructed the phylogenetic relationships of exon 2 MHC IIB of all alleles we retrieved in the Midas cichlid, and 20 alleles of several other fish species of cichlids and non-cichlids (*Sebastes caurinus*, *Sebastes maliger*, *Miichthys miiuy*, *Pundamilia nyererei*, *Haplochromis sp. ‘rockkribensis’*, *Haplochromis xenognathus*, and *Oreochromis niloticus* (see Additional file 1: Table S3)). All of these alleles clustered within the Midas cichlid alleles providing evidence for trans-species polymorphism. Nile tilapia (*Oreochromis niloticus*), the species for which there exists most MHC information, had alleles distributed throughout the tree (Fig. 5). One of these alleles clustered with our Group I (DJB accession # AB677258.1). Several alleles from different species clustered with alleles in Group IV, and also with the ungrouped alleles.

### Test of selection

The analyses of site-specific selection revealed that alleles in groups II, III and IV had sites that fitted best the model with positive selection (Group II, *p* = 0.02; groups III and IV, *p* < 0.001), while alleles in Group I did not (*p* = 0.10). Alleles in Group I did not have any synonymous substitutions, therefore d_N_ / d_S_ ratios could not be calculated. We tested all alleles including those in Group I, and found 19 positively selected sites, 13 of which were inferred at 99% level (Table 3). When we excluded alleles in Group I, 19 and 20 sites were inferred to be positively selected for models M2a and M8 respectively, 15 and 16 of them were inferred at 99% level. However, only 4 sites overlapped with those in the previous analysis. For the groups II, III and IV, we found between 1 and 9 sites to be under positive selection (Table 3). However, these sites were not shared between groups whether or not Group I was included, indicating that the groups might have followed different evolutionary trajectories.

**Table 3:** Summary of likelihood ratio tests for site-specific positive selection of MHC IIB genes comparing groups of alleles

Allele Group	Models compared	LRT (2Δl)	p-value	Estimate for ω >1	Proportion of PSS	PSS
All groups	M1a versus M2a	81.738836	1.09E-06	3.71226	0.34292	**1**, **2**, 7, 9, **10**, **32**, 33, **37**, **38**, **39**, 41, **42**, **43**, 46, **57**, **58**, **60**, 61, **62**
All groups	M7 versus M8	84.941740	6.61E-07	3.55150	0.35599	**1**, **2**, **7**, 9, **10**, **32**, **33**, **37**, **38**, **39**, **41**, **42**, **43**, **46**, **57**, **58**, **60**, 61, **62**
All groups	M8a versus M8	73.238718	6.12E-07			
All except I	M1a versus M2a	83.327546	1.16E-06	3.89788	0.32156	**8**, **9**, **16**, **17**, 32, 39, **40**, **44**, **45**, **46**, 48, **49**, **50**, **53**, **64**, **65**, **67**, 68, **69**
All except I	M7 versus M8	80.631206	6.74E-07	3.72628	0.33118	**8**, **9**, 14, **16**, **17**, 32, **39**, **40**, **44**, **45**, **46**, 48, **49**, **50**, **53**, **64**, **65**, **67**, 68, **69**
All except I	M8a versus M8	74.046658	8.90E-07			
Group I only	M1a versus M2a	4.594664	0.10			
Group I only	M7 versus M8	4.594238	0.10			
Group I only	M8a versus M8	4.594000	0.10			
Group II only	M1a versus M2a	7.702834	0.021	3.16267	0.48401	49, 65
Group II only	M7 versus M8	7.706184	0.021	3.16268	0.48401	13, 49, **65**
Group II only	M8a versus M8	7.702818	0.021			
Group III only	M1a versus M2a	14.605000	0.0007	31.27408	0.03609	**73**
Group III only	M7 versus M8	14.663852	0.0007	31.37037	0.03603	**73**
Group III only	M8a versus M8	14.604918	0.0007			
Group IV only	M1a versus M2a	20.610518	0.00003	7.27232	0.15698	11, **48**, **55**, 67, **69**, **106**
Group IV only	M7 versus M8	20.981230	0.00003	7.22462	0.15872	**11**, 42, **48**, **55**, 66, 67, **69**, 77, **106**

The LRT models compared by the likelihood ratio test with five codon substitution models: Beta models M7 (no positive selection), M8 (positive selection), and M8a (no positive selection and ω = 1), and models M1a (nearly neutral) and M2a (positive selection). The 2Δl = 2(lb - la), ω = d_N_ / d_S_, positively selected sites (PSS) are inferred by empirical Bayesian posterior probabilities. PSS in bold are inferred at 99%

The tests of overall selection among all alleles showed that the entire sequence (LP, β_1_ and β_2_ domains) of all alleles is under purifying selection (*p* = 0.021), and this pattern remains when the alleles of Group I are excluded (*p* = 0.005) (Table 4). Nine alleles were excluded from the analysis of the β_2_ domain because we did not obtain sequences of this domain for those alleles. The β_2_ domain shows purifying selection for all alleles (*p* < 0.001), as well as when excluding Group I (*p* = 0.001). However, neither the β_1_ domain, nor the entire sequence showed signs of overall positive selection (*p* = 1.00) excluding Group I (*p* = 0.001) (Table 4).

**Table 4:** Tests of overall selection and selection by domain

Domain	Alleles	length (AAs)	dN	dN Number	dS	dS Number	dN/dS	*P-* value (purifying)	*P-* value (positive)
LP, β1 & β2	Group I-IV and ungrouped (N69)	228	0.221 (0.024)	44.488 (4.998)	0.327 (0.053)	18.525 (2.234)	0.676	**0.021**	1.000
β1	Group I-IV and ungrouped (N69)	91	0.254 (0.032)	37.703 (3.988)	0.301 (0.059)	12.807 (1.679)	0.844	0.219	1.000
β2	Group I-IV and ungrouped (N60)	93	0.129 (0.029)	8.502 (2.154)	0.459 (0.101)	7.238 (1.585)	0.282	**<0.001**	1.000
β1 & β2	Group I (N20)	113	0.008 (0.003)	1.616 (0.521)	0.000 (0.000)	0.000 (0.000)	-	-	-
LP, β1 & β2	Group II-IV and ungrouped (N49)	229	0.193 (0.022)	41.637 (4.845)	0.306 (0.044)	18.101 (2.142)	0.633	**0.005**	1.000
β1	Group II-IV and ungrouped (N49)	91	0.216 (0.028)	33.987 (3.776)	0.270 (0.049)	11.568 (1.579)	0.800	0.131	1.000
β2	Group II-IV and ungrouped (N40)	94	0.138 (0.034)	9.378 (2.293)	0.463 (0.114)	8.030 (1.679)	0.298	**0.001**	1.000

Tests of overall selection on the entire length of sequences, and on the different domains were performed. The leading peptide LP, and the two domains β_1_ and β_2,_ comprise the entire known sequence, despite not all alleles have full coverage of both domains and the LP. The amino acid lengths for each test are given as the number of sites that were included in the test. Three alleles (0005, 0006 and 0012) from Group I, and six alleles (0203, 040103, 0406, 05, 25, 27) from Groups II-IV were excluded for the test of selection on the β_2_ domain, as they lack sequence information for this domain. d_N,_ number of non-synonymous substitution per non-synonymous site; d_S,_ number of synonymous substitutions per synonymous site; standard error (SE). Significant p-values (at 99% confidence level) are shown bold

### Protein structure homology models

We built protein homology models for all alleles to characterize their tertiary structure, and to determine if they can fold into a potentially functional MHC molecule. The QMEAN4 ranged between -1.72 and -4.32, indicating generally good quality of the proposed models (see Additional file 1: Table S6 for details). GMQE score ranged between 0.61 and 0.75, indicating an overall good quality for most models (Additional file 1: Table S6).

**Fig. 6:**
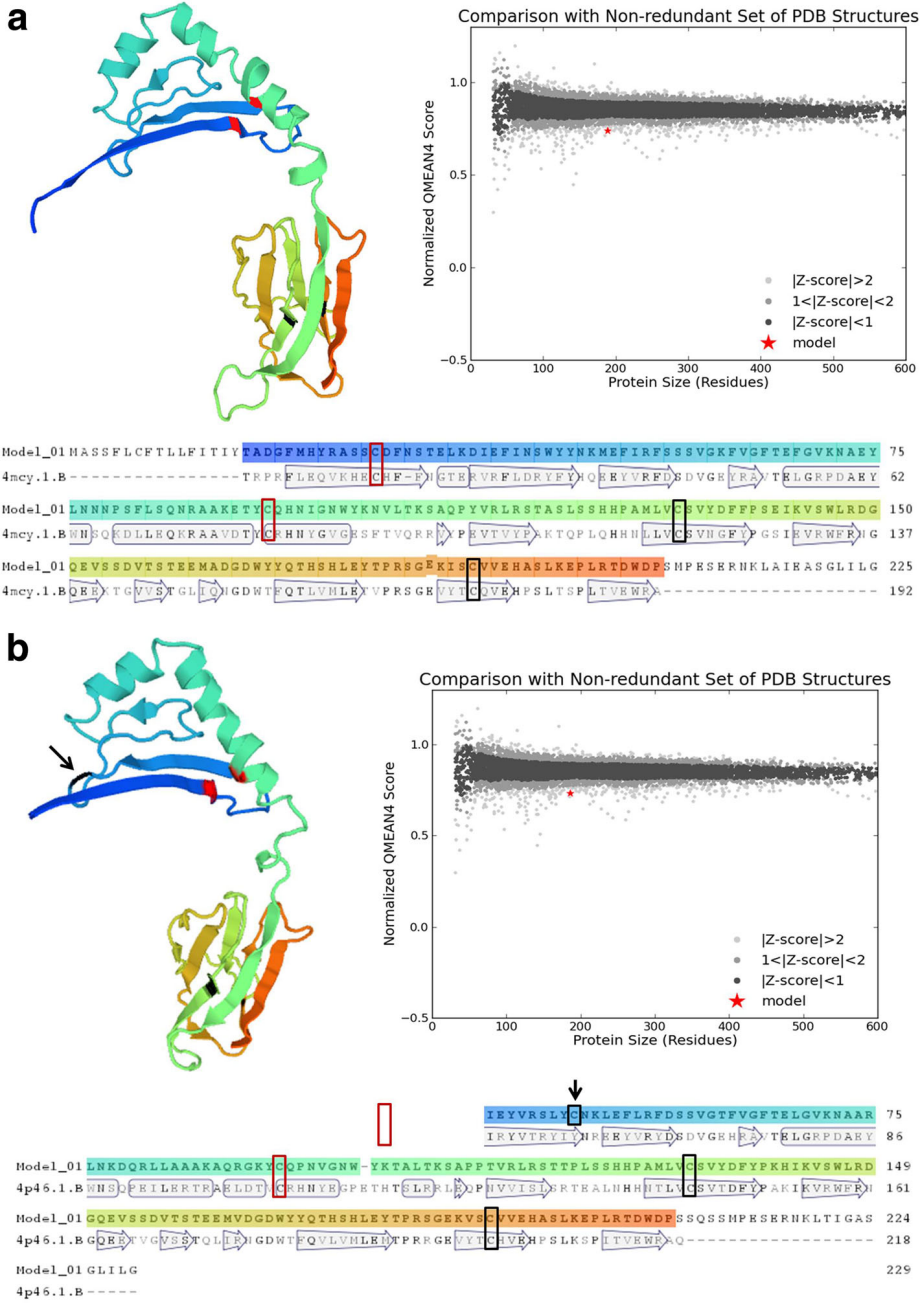
Models of tertiary structure of MHC IIB sequences, where red boxes represent cysteine amino acids forming the disulfide bond in the β_1_ domain, and black boxes represent cysteine residuals that form the disulfide bonds of the β_2_ domain. **a** Model of allele DXB*2202; **b** Model of allele DXB*060101, with an unpaired cysteine at position 47 (indicated with a black arrow). The graphs show how each model (red star) compares to a non-redundant set of Protein data bank (PDB) structures, indicating the quality of the model compared to molecules of the same size as a value equivalent to a z-score test

We located the cysteine residues in the 3D structure of alleles to see if they were in the correct position to make structural disulfide bonds. All alleles had an unpaired cysteine at position 7 of the leader peptide, and the four expected cysteine residues at positions 29, 94, and 132, 188, which pair covalently to form disulfide bonds that increase the stability of the β_1_ and β_2_ subunits respectively (Fig. 6a). All alleles in Group III had an additional unpaired cysteine at position 47 (Fig. 6b), and all alleles in Group II-b had an additional cysteine at position 98. Alleles of Group I showed no notable anomalies in their 3D structure, but allele DXB*0007 presented an unpaired cysteine at position 47 similarly to alleles of Group III. The N-terminal area of MHC IIB protein included an alpha helical region and a beta sheet of four strands in antiparallel orientation. It also showed that the C-terminal area mainly has a beta-fold structure and is characterized by an immunoglobulin-like beta-sandwich made of two anti-parallel sheets. Interestingly, our work revealed that all 3-D models were similar among all groups of alleles, with the exception of the unpaired cysteine positions.

## Discussion

We found a total of 69 alleles of MHC IIB exons 2 and 3 in 13 Midas cichlid individuals. This represents very high allelic diversity in this species despite the small sample size tested. Individual Midas cichlids harbor a large number of alleles, with a maximum of 25 per individual, and an average of 12.5 (SD = 6.1). Together, this implies that the Midas cichlid has at least 13 distinct MHC IIB loci, although this may be an underestimation as we cannot detect allele sharing between loci, and we may have insufficient sequences for some alleles. In other Old World cichlid species, up to 17 polymorphic loci have been found, and between 1–13 alleles per individual [51]. Hence, our characterization of Neotropical cichlids revealed comparable structure and diversity between Old and New World cichlids.

Large variation in number of MHC loci, total number of alleles at the population level, and heterozygosity at the individual level, have been reported among fish taxa. For example, pipefish (*Syngnathus typhle*) and Atlantic cod (*Gadus morhua*) have lost all MHC class II loci [73, 74], and appear to compensate for it by larger diversification of MHC class I [75]. Seahorse (*Hippocampus abdominalis)* has a single MHC IIB locus, and a maximum of two alleles per individual [76]. Spotted halibut (*Verasper variegatus*) and Japanese flounder (*Paralichthys olivaceus)* have up to five alleles per individual [77, 78], and guppy (*Poecilia reticulata*) and eel (*Anguilla anguilla*) have up to six alleles [59, 79], which suggests that they all have at least 3 distinct loci. Stickleback (*Gasterosteus aculeatus*), a species with thoroughly studied MHC, has between 3 and 6 MHC class IIB loci depending on the population of origin [57, 80]. However, cichlid MHC IIB alleles are more numerous than those of any other fish that has been studied. This may have contributed to, or be a result of their great diversification.

Because MHC genes are directly responding to local parasite pressure [81, 82] they may encode for a magic trait contributing to local adaptation and ultimately to ecological speciation [14, 83]. Determining the contemporary MHC diversity is however challenging due to its multigene nature and fast turnover, often affecting conserved regions that are typically used for setting up primers for amplification [84].

Neotropical cichlids are model species for speciation in sympatry [28], but their MHC genes had not been characterized to date. The nearest relatives with well characterized MHC class II genes are African cichlids [50, 70, 85], from which they split 93 MY ago [54]. We therefore carried out this study to establish reference sequences in order to obtain specific primers that would amplify a comprehensive diversity of MHC IIB genes for future population-based studies.

Like most other fish, the sequences we obtained of the Midas cichlid MHC IIB genes are composed of 6 exons [5, 85]. We did not obtain the sequence of all exons for all alleles, but for those we have the complete sequence there are always 6 exons. Published sequences of these genes in other cichlids show the same intron-exon organization [5, 85]. This is the most standard structure in fish, even though species with 5 exons and functional MHC have also been described (e.g., sea bream *Chrysophrys major* [86] or Japanese flounder *Paralichthys olivaceus* [87])*.* Variation in exon number is due to either exon fusion [88], or exon splitting [5, 89].

We did however find considerable variation in the length of intron 2, which ranged from 239 bp to 2.5 kb in the sequences we obtained, and 10 kb in the genome sequences. This intron length variation is likely the reason why we were unable to amplify some alleles that were obtained from cDNA when using the same primers spanning introns in gDNA. Length variation in intron 2 has been reported in several species including other cichlids [51, 90, 91]. The tandem repeats found in intron 2 contribute to the increased length of the long intron. Reusch and Langefors [7] reported a 10-mer repeat in intron 2 of three-spined sticklebacks, responsible for important changes in sequence length, demonstrating that this mode of intron evolution can happen in other fish species. The genome sequences also revealed length variation in all other introns, most notably in intron 3 that varies between 155 to 5 kb.

Within the 69 alleles found in the Midas cichlid we distinguished different groups. One group of alleles (Group I) resembled a pattern of non-classical MHC IIB genes [92]. These alleles showed low variability, are apparently not expressed, and none of their positions seemed to have evolved under positive selection according to our selection analyses. A pattern of low polymorphism is typical of non-classical MHC IIB genes, since their primary function is to assist in loading the antigenic peptides onto classic MHC class II molecules [93]. Because non-classical MHC molecules do not have to bind to a wide array of antigen peptides, the sequence between the different alleles do not follow the typical “patchwork” pattern of classic MHC II alleles, especially in the peptide binding region [93]. Non-classical MHC IIB genes similar to those in Group I of the Midas cichlid have also been described in Atlantic Salmon (*Salmo salar*) [94]. All other groups of alleles and the ungrouped alleles showed classic MHC class II gene patterns. They all displayed sites subjected to strong positive selection, suggesting that they might have evolved under strong parasite-mediated balancing selection [95, 96]. However, the results of the selection tests have to be taken with caution since we cannot allocate alleles to specific loci and alleles within groups could potentially belong to different evolutionary lineages. This might also have an influence on the overall selection tests for which all groups were combined. It might explain some of the discrepancies between strong positive selection in site-specific tests and the absence of positive selection in the overall tests. Antagonistic coevolution between hosts and parasites is recognized as a powerful force capable of driving rapid evolutionary changes, which might significantly contribute to biodiversity (e.g., [91]). In fish, MHC frequency shifts of resistance alleles have been observed as a response to local parasite-mediated selection [83]. Combined with MHC-based mate choice reported in almost all jawed vertebrates [82], host-parasite interaction through MHC genes has been suggested to contribute to speciation, even in sympatry [37, 97–99].

The phylogenetic tree of all 69 alleles plus 20 of other fish species, displayed a pattern that strongly supports trans-species polymorphism. Some alleles of the Midas cichlid seem to be more closely related to alleles of other species than to other alleles of the same individual. For example, alleles of Group I are more closely linked to Nile tilapia DJB allele (DJB accession # AB677258.1) than to any other Midas MHC IIB allele. Similarly allele DXB*27 of the Midas cichlid is closely related to allele DIB of Nile tilapia, indicating homology and hence TSP. TSP is a common pattern of the MHC and has been observed in many taxonomic groups (reptiles [42], amphibians [44], mammals [43], birds [45, 46], and fish [39, 47]). TSP is evident throughout the phylogenetic tree, and seemed to be most common with alleles of Group IV and ungrouped alleles.

The tertiary structure models showed that similar to the Nile tilapia [100], the Midas cichlid MHC IIB sequence has all the necessary features for the molecule to be functional, including two pairs of cysteine residues. The biological function of unpaired cysteine residues in the MHC molecules remains unknown. It has however been suggested that they could play a role in the formation of exosomal dimers [101]. We found two groups of alleles with extra unpaired cysteines (groups II-b and III), but nothing noteworthy was found in the structure of these alleles. Future studies focusing on the tertiary structure of MHC molecules should focus on determining the function of unpaired cysteines, to further reveal their contribution to immunity specifically, and species’ evolution in general.

Despite considerable sequencing effort we were not able to find all alleles in both cDNA and gDNA. Alleles in Group I were never detected in cDNA, which makes us think they might be putative non-classical or even pseudogenes. On the other hand, we had difficulties in amplifying alleles from Group III in gDNA while they were readily obtained in cDNA. We only succeeded in amplifying these alleles in gDNA by using primers that excluded intron 2. Intron 2 is therefore likely causing sequencing difficulties due to particularly long sequences or rich GC content. Another explanation for these difficulties might be alternative splicing, which is known to occur in MHC [102]. Indeed, in salamanders over 20% of the transcripts can be alternatively spliced, with variation in different organs, see Bulut et al. [103]. As the alleles discovered here seem to be functional and variable, and they may be contributing to the dynamic response of MHC to parasite selection [99].

## Conclusions

Taken altogether, MHC IIB genes in the Midas cichlid showed enormous richness in allele diversity and copy number. This diversity is larger than that described in most other fish, and is only comparable to that found in other cichlids. Our findings promise great potential in studying the processes of evolution and speciation in this model system and should be further studied at the ecotype, population and species levels to elucidate the role that parasites may play in sympatric speciation.

## Additional file

Additional file 1: Figure S1. Aminoacid variability of all obtained MHC IIB sequences. **Figure S2.** Phylogenetic inference tree of MHC IIB alleles. **Figure S3.** Estimate of evolutionary divergence between sequences that support allele groupings. **Table S1.** List of samples used for this study, Species, Lake and ID numbers are given. **Table S2.** List of sequences from GeneBank used to design primers MHC-Rev_3. **Table S3.** Sequences of diferent species used to evaluate trans-species polymorphisim. **Table S4.** Length in base pairs for the exons and Introns sequenced for each allele and *O. niloticus* sequence used as reference. **Table S5.** Mean pairwise distances of randomization analysis on the groups of alleles. **Table S6.** 3D Protein homology models for all alleles with the global model quality estimation, the overall model quality scores, and the summary of estimated Z-scores. (DOCX 8379 kb)

## Abbreviations

cDNA: Complementary DNA
gDNA: Genomic DNA
MHC: Major Histocompatibility Complex
TSP: Trans-species polymorphism
