# Supplementary material for: Molecular characterization of MHC class IIB genes of sympatric Neotropical cichlids
Source: BMC Genet. 2017 Feb 15;18:15. doi: 10.1186/s12863-017-0474-x (PMC5310070; doi:10.1186/s12863-017-0474-x)
Supplement: Additional file 1: Figure S1. — Aminoacid variability of all obtained MHC IIB sequences. Figure S2. Phylogenetic inference tree of MHC IIB alleles. Figure S3. Estimate of evolutionary divergence between sequences that support allele groupings. Table S1. List of samples used for this study, Species, Lake and ID numbers are given. Table S2. List of sequences from GeneBank used to design primers MHC-Rev_3. Table S3. Sequences of diferent species used to evaluate trans-species polymorphisim. Table S4. Length in base pairs for the exons and Introns sequenced for each allele and O. niloticus sequence used as reference. Table S5. Mean pairwise distances of randomization analysis on the groups of alleles. Table S6. 3D Protein homology models for all alleles with the global model quality estimation, the overall model quality scores, and the summary of estimated Z-scores. (DOCX 8379 kb) [file 12863_2017_474_MOESM1_ESM.docx]

**Supplementary Tables and Figures**

**
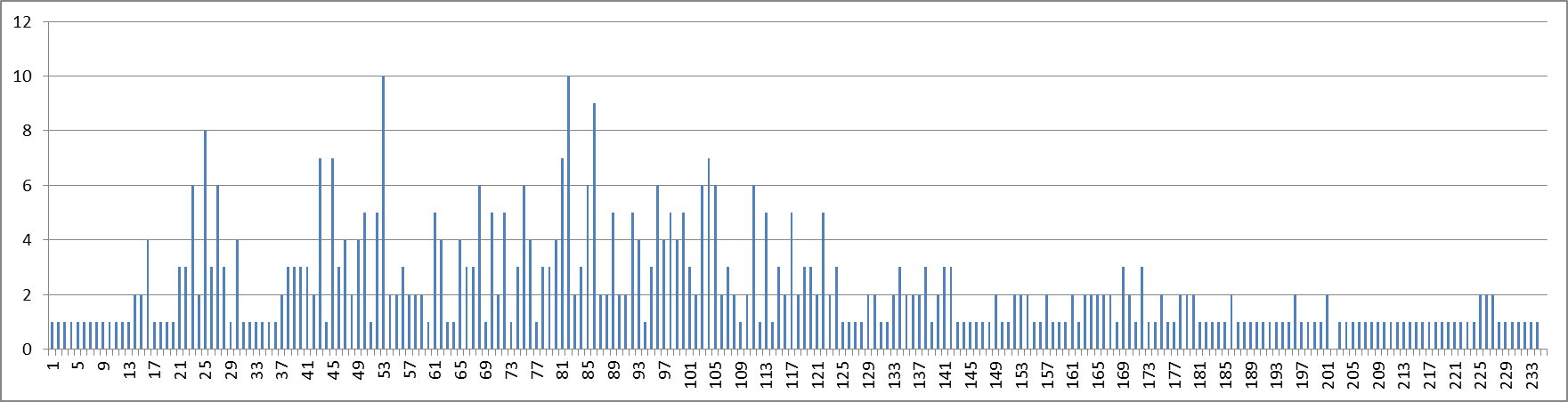
Sup. Fig. 1.** Aminoacid variability ranging from 1-10 aminoacids per site.

**Sup. Fig. 2.** Phylogenetic inference relationship of all MHC IIB alleles with posterior probabilities shown for each node.


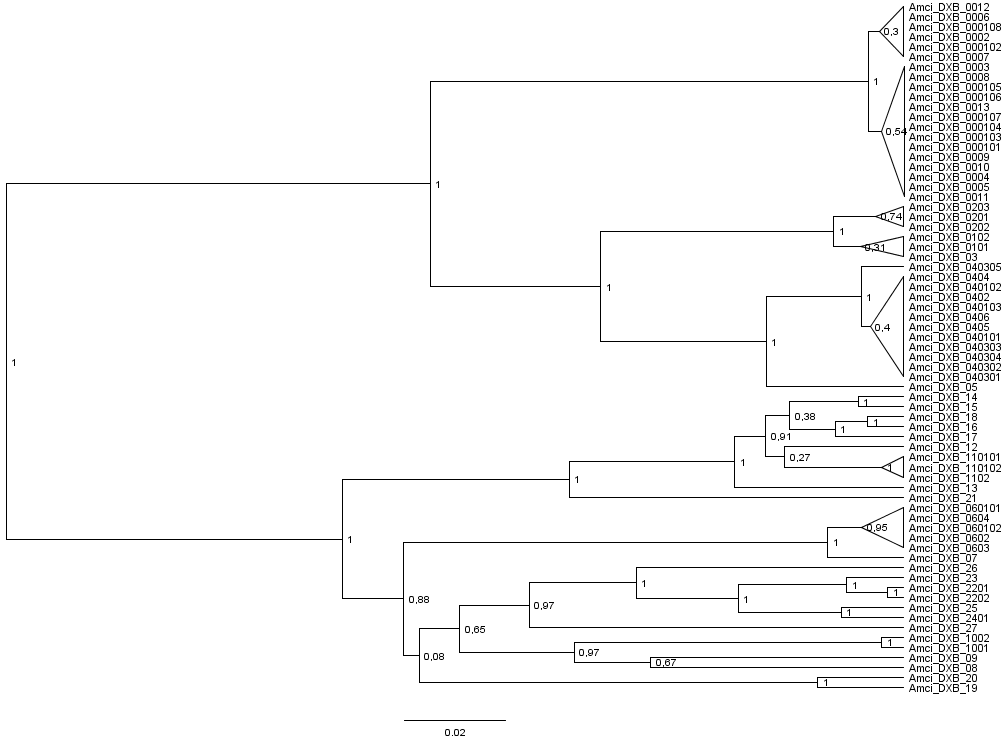


**Suppl. Fig. 3.** Estimates of evolutionary divergence between sequences. The number of base substitutions per site between sequences are shown.

**Sup. Table 1.** List of samples used for this study, with their ID, species,, lake of origin and whether we obtained gDNA, cDNA or both.

**Sup. Table 2.** List of sequences used to design primers “MHC-Rev_3” all obtained from NCBI´s GeneBank.

**Sup. Table 3.** List of sequences of MHC IIB spanning exon 2 and 3 used from different fish species obtained from the literature to evaluate trans-species polymorphisim.

**Sup. Table 4.** List of the alleles found in this study with the length in bp for each exon and intron. On the bottom are provided for reference the lengths of the elements of an allele of *O. niloticus* for which there is a complete reference sequence (Accession no.: JN967618). Exon (E) and Intron (I). Bold (complete sequence) italic (partial sequence).

**Sup. Table. 5**. Randomization analysis on the groups of alleles estimated with the estimates of evolutionary divergence between sequences. P-values were estimated with 999 randomizations.

**Sup. Table 6 and Figures of 3D Models.** Protein homology models for all alleles were built with Swiss-Model workspace v8.05 (Arnold et al. 2006). We report the templet used to build each model, the percent Identity with said templet, the global model quality estimation (GMQE), and the overall model quality (QMEAN4). The Summary of normalized QMean4 scores of all 3D Homology models compared with a non-reduntand set of Protein Data Bank Bank(PDB) structures to infere Z-scores is also reported.

Amci-DXB*000101


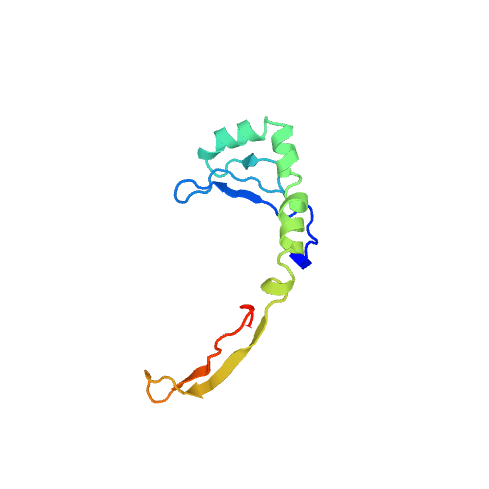

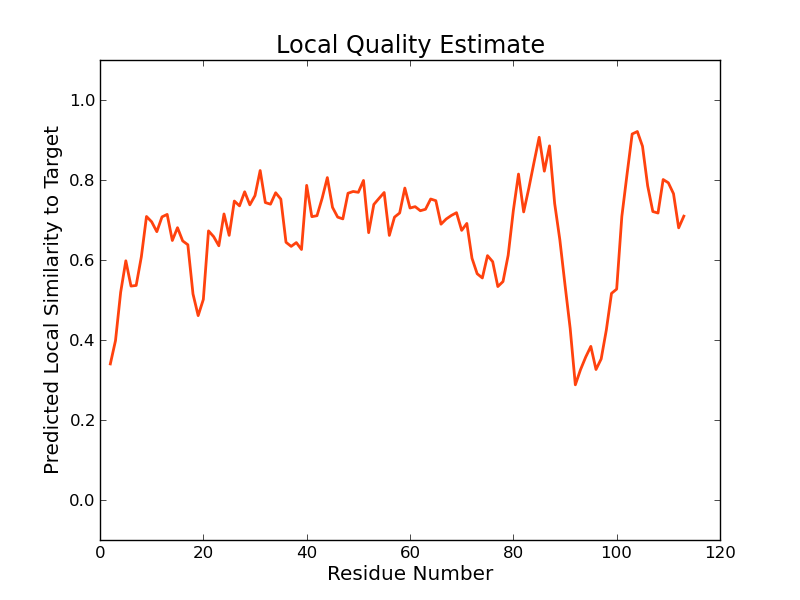


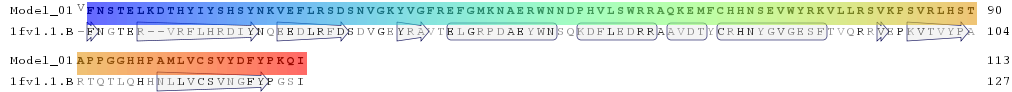


Amci-DXB*000102


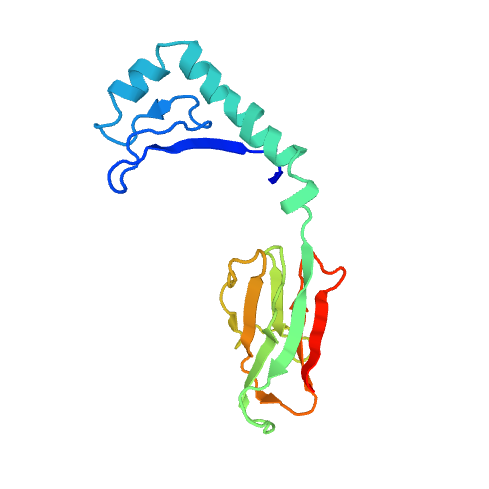

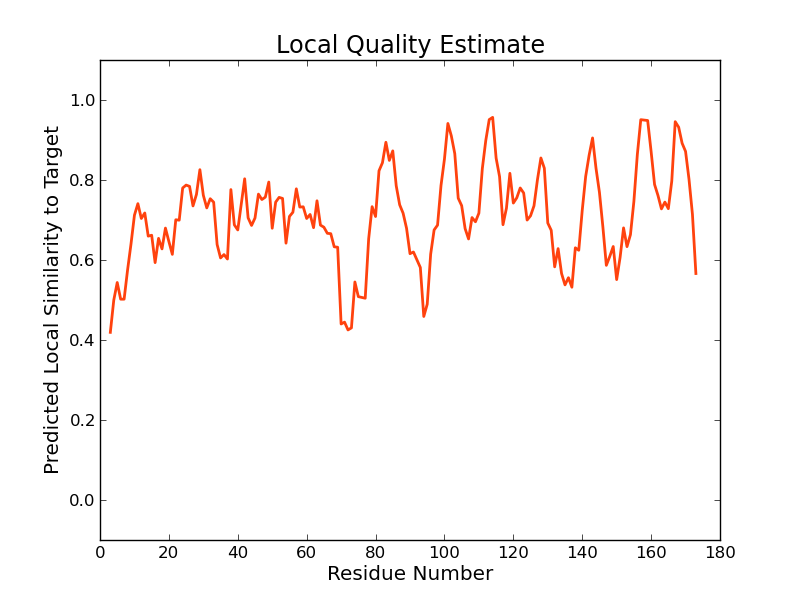


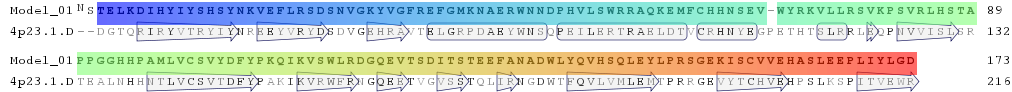


Amci-DXB*000103


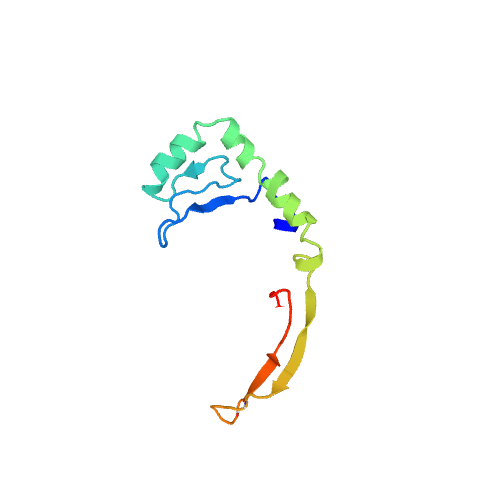

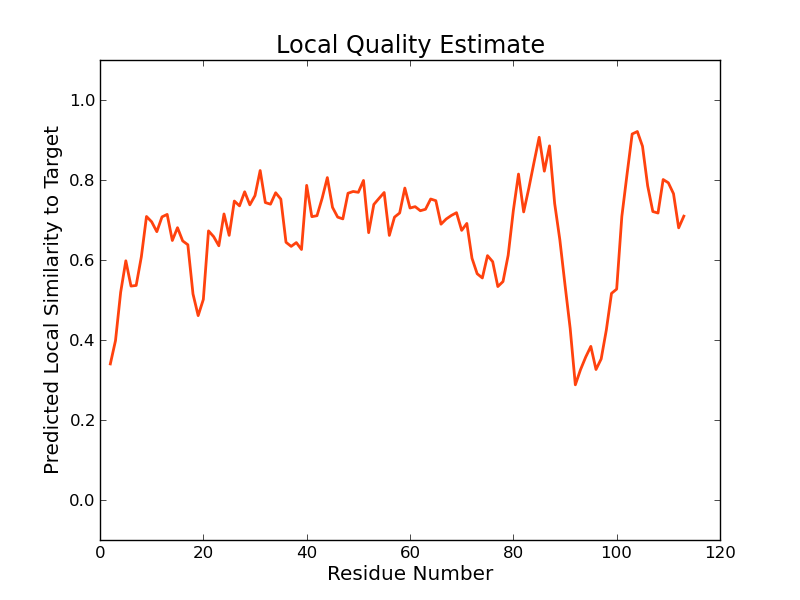

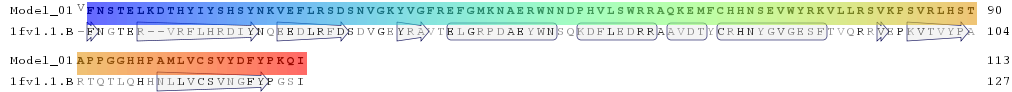


Amci-DXB*000104


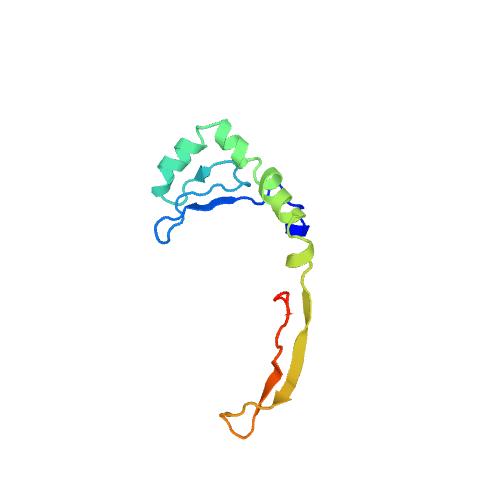

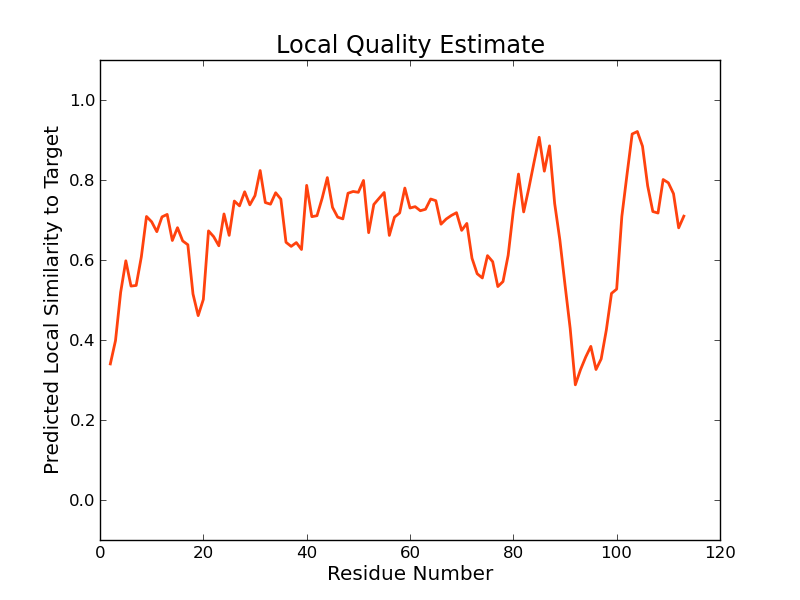

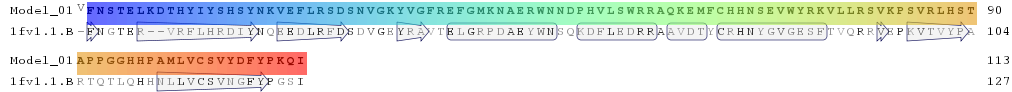


Amci-DXB*000105


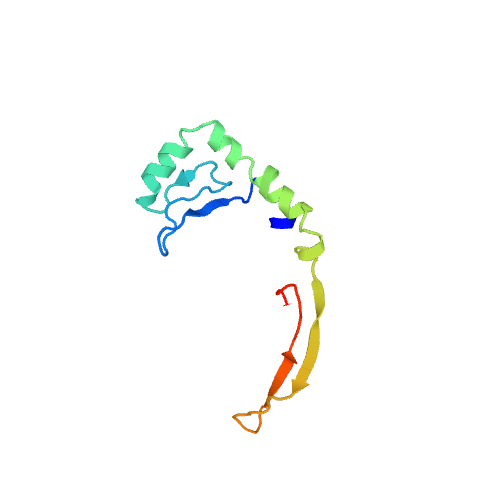

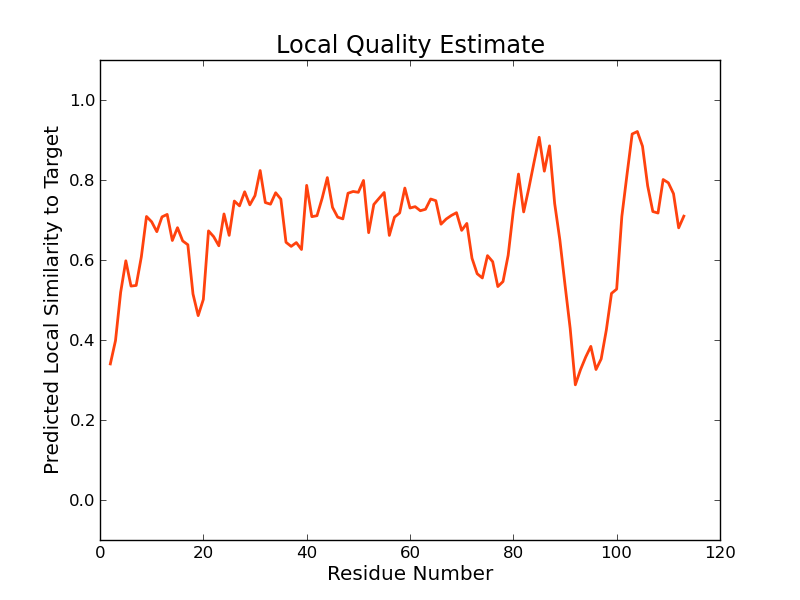

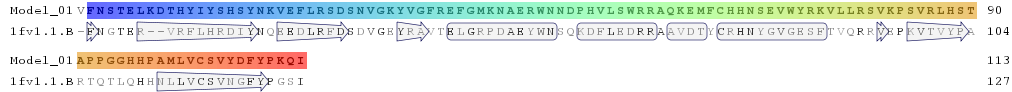


Amci-DXB*000106


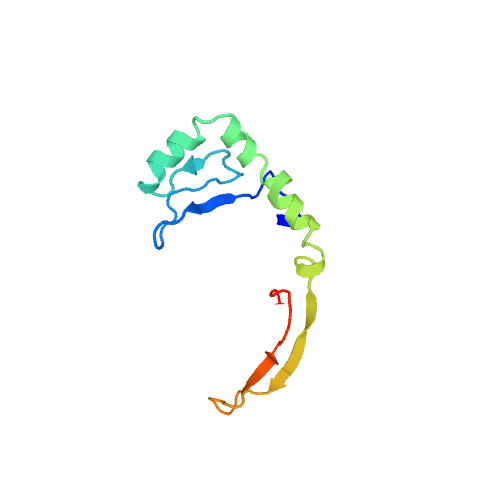

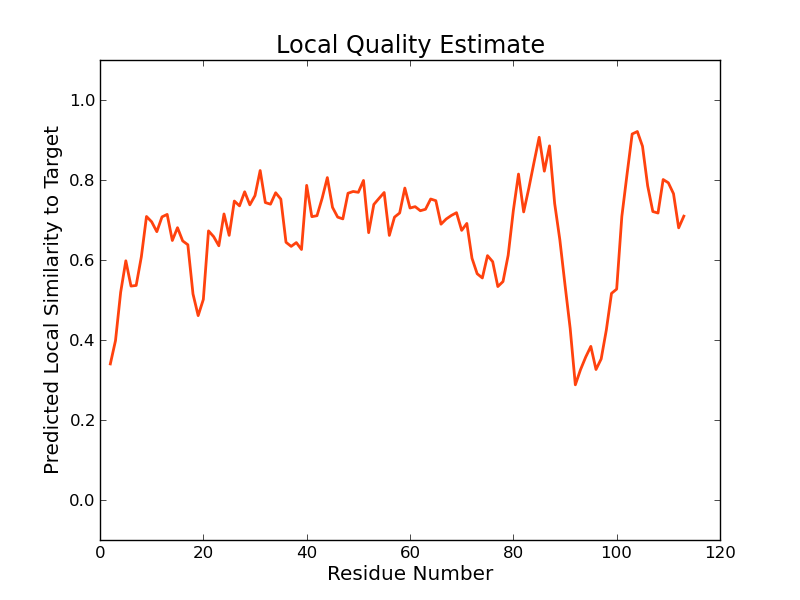

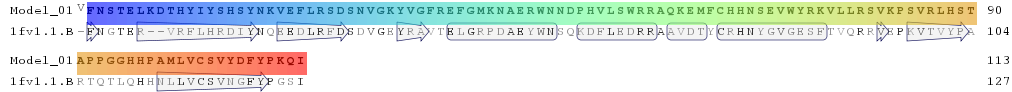


Amci-DXB*000107


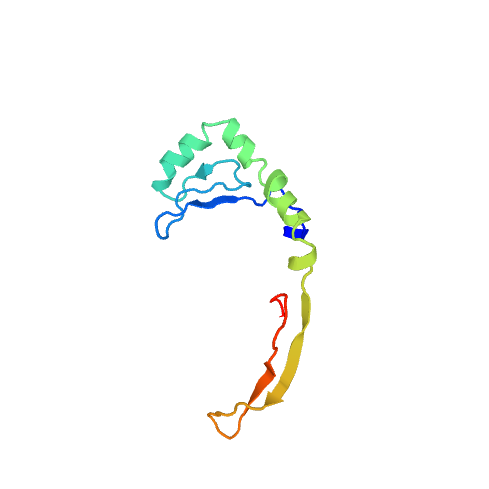

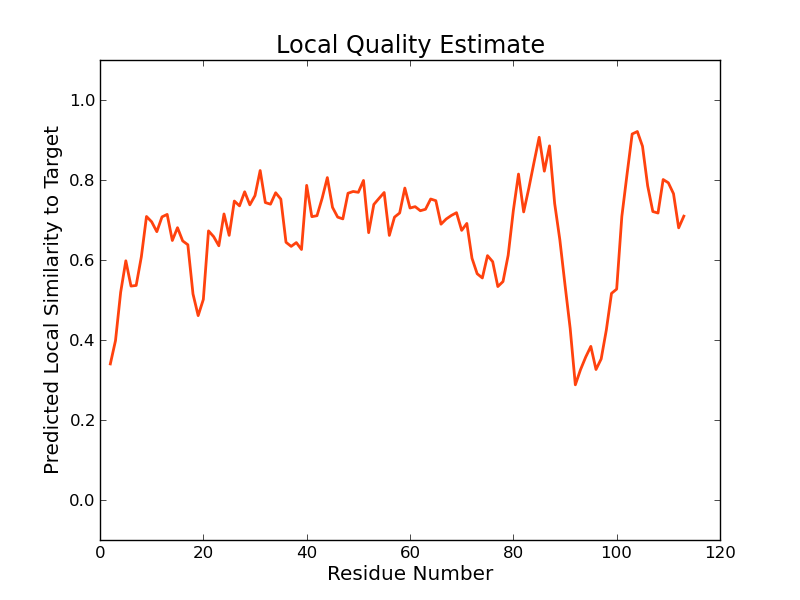

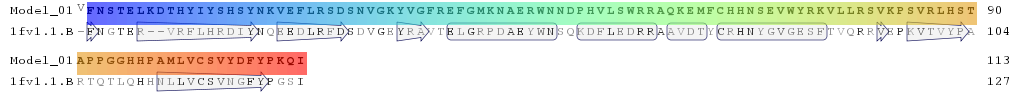


Amci-DXB*000108


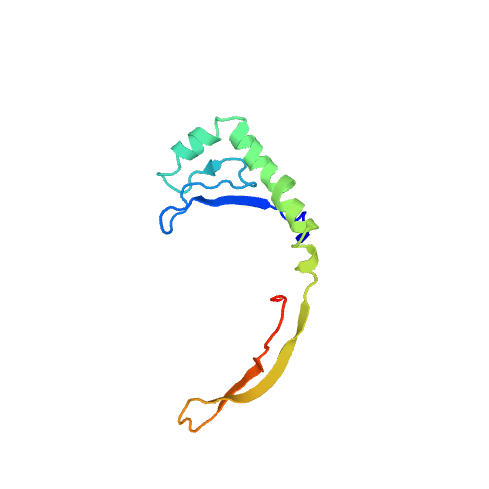

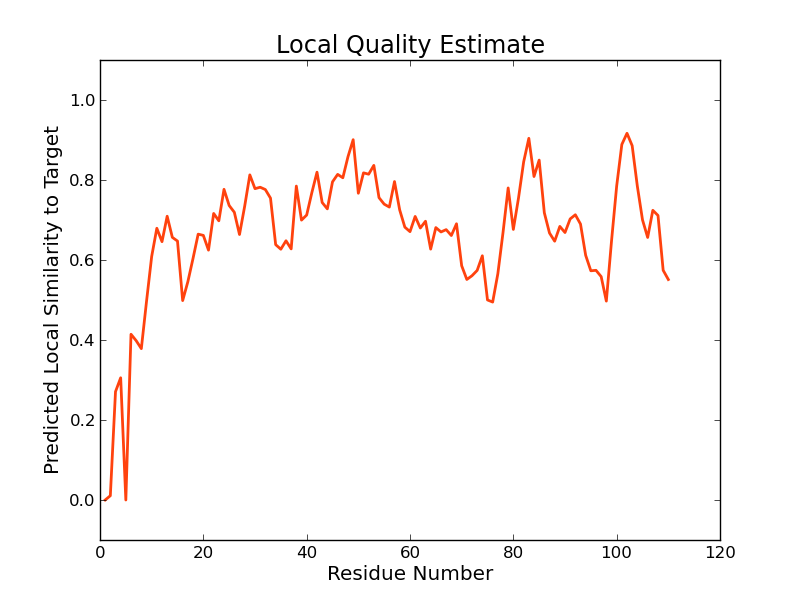


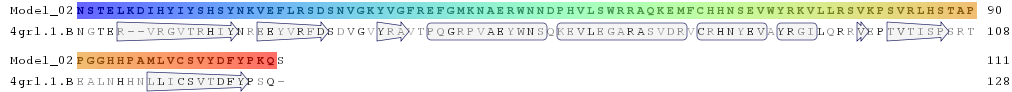


Amci-DXB*0002


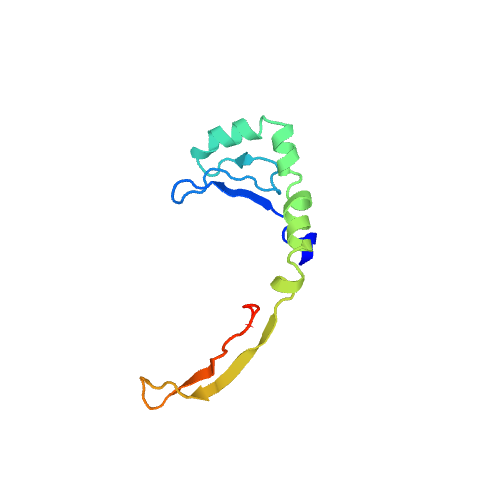

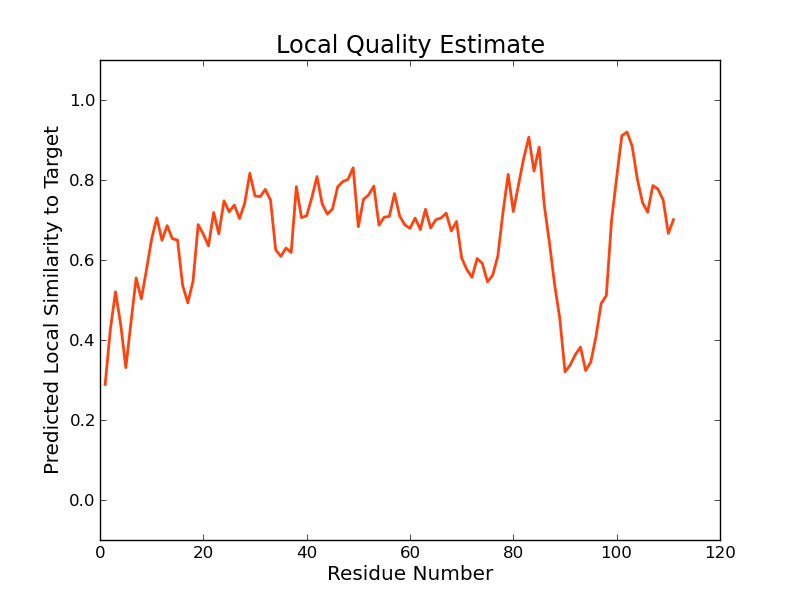


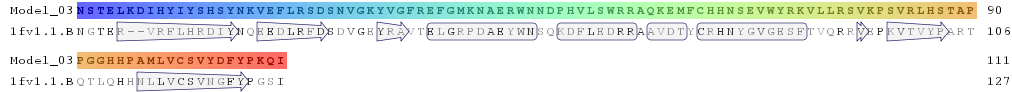


Amci-DXB*0003


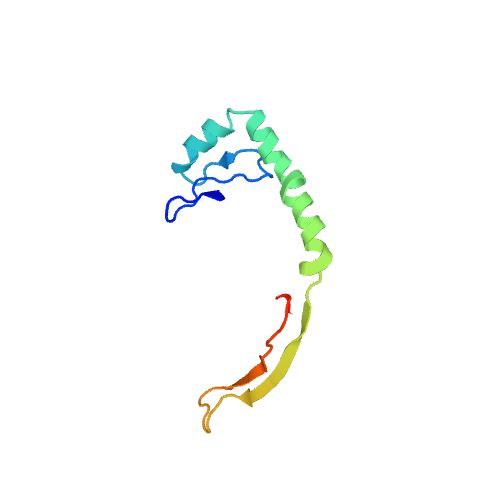

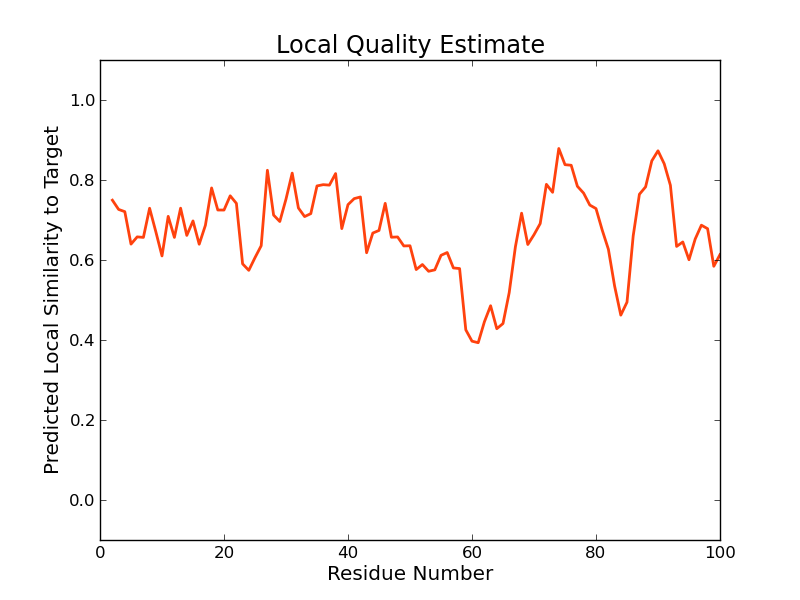

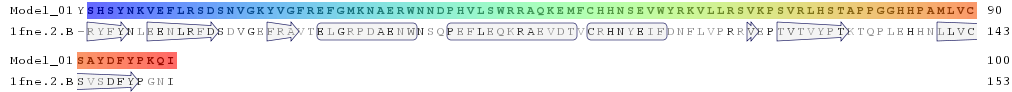


Amci-DXB*0004


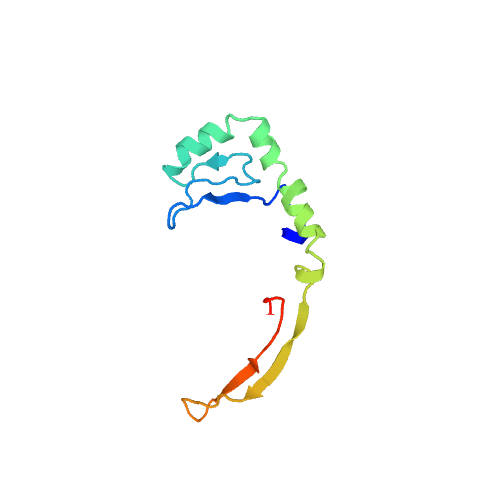

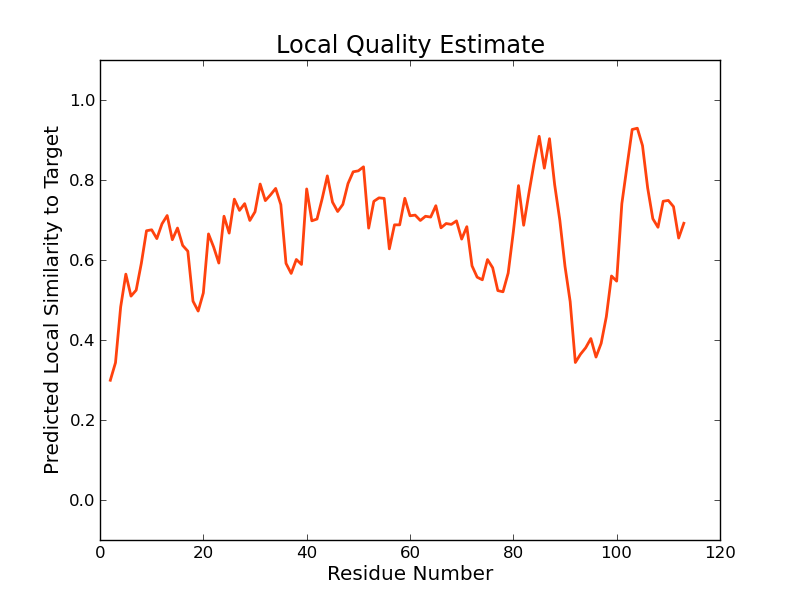


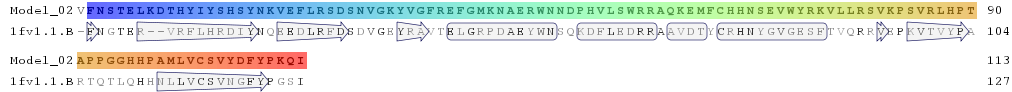


Amci-DXB*0005


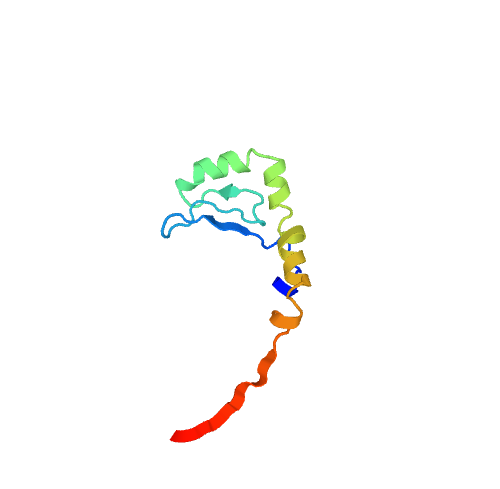

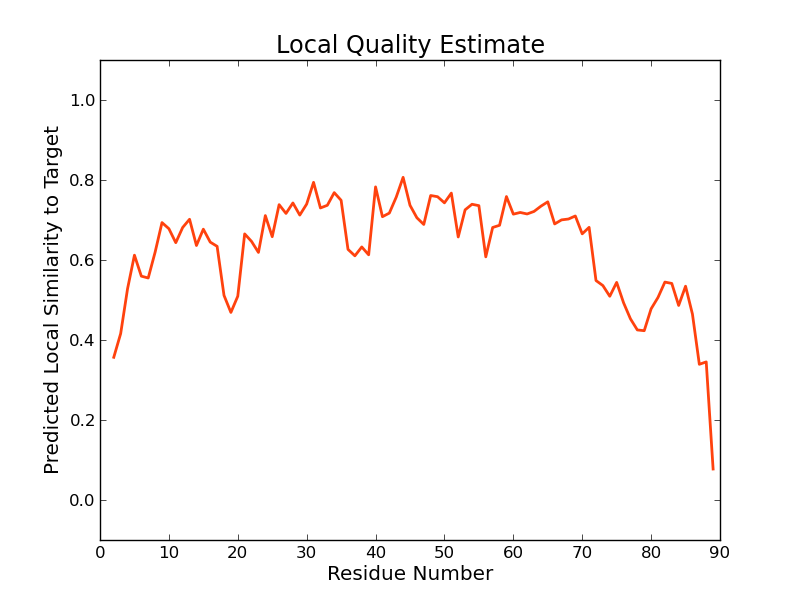

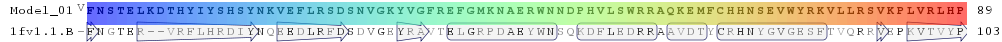


Amci-DXB*0006


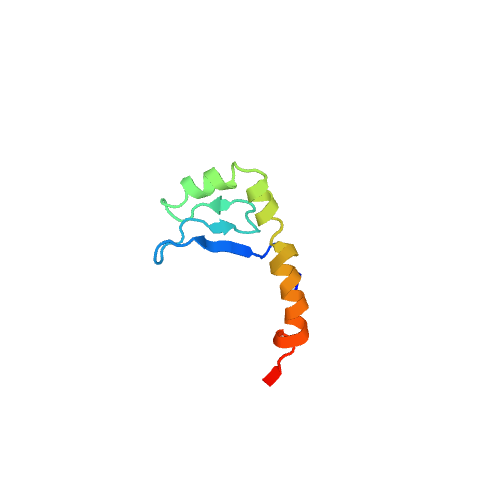

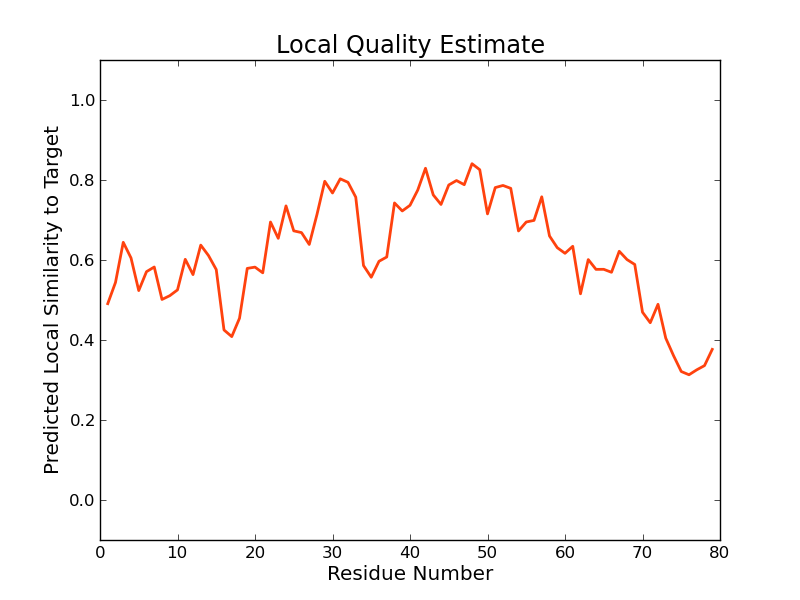


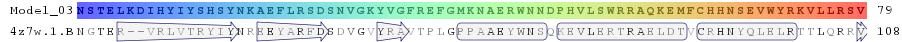


Amci-DXB*0007


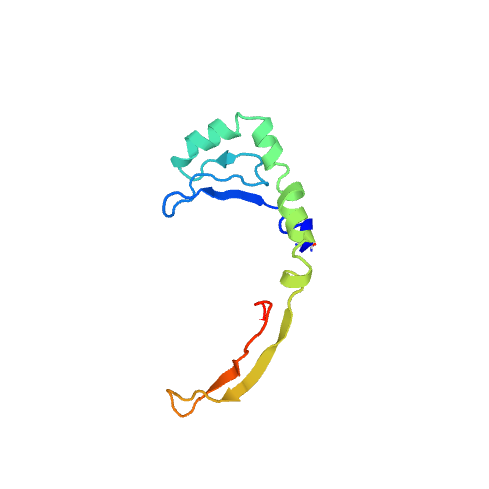

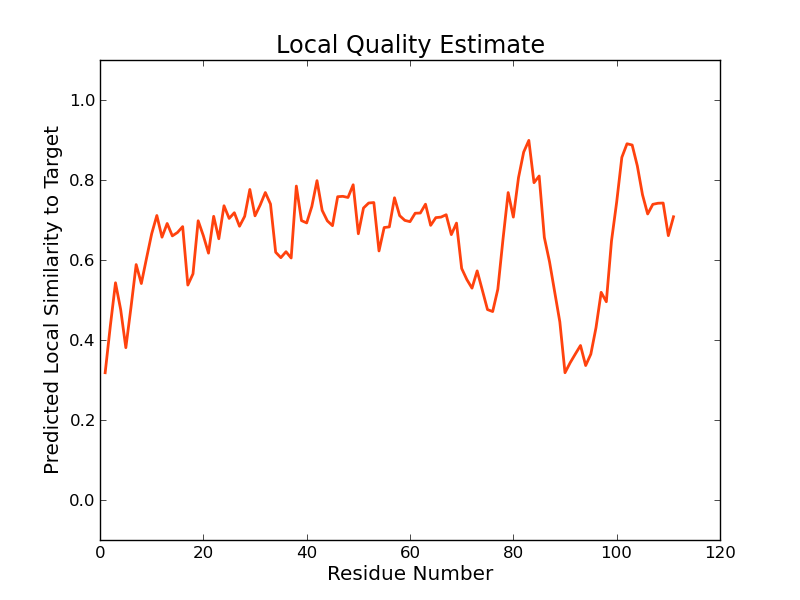


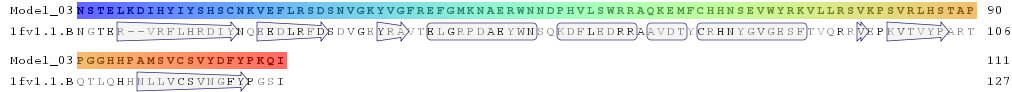


Amci-DXB*0008


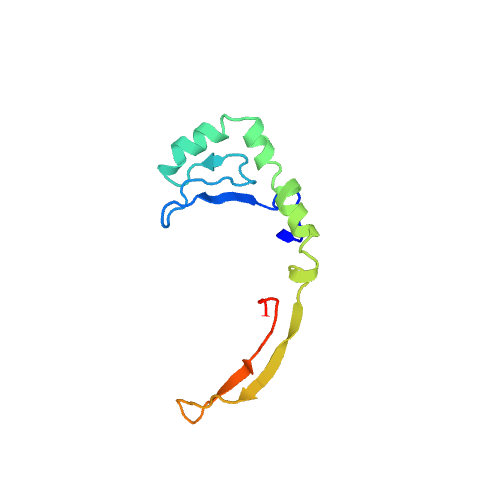

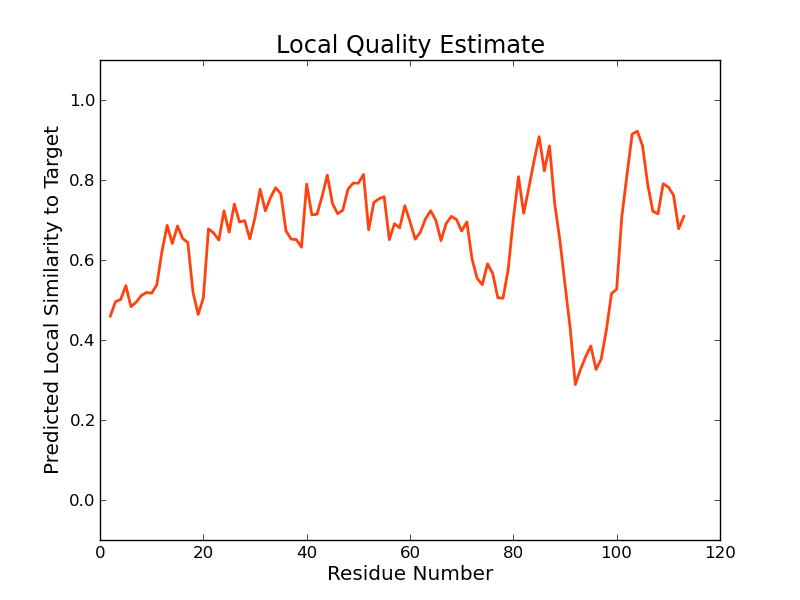


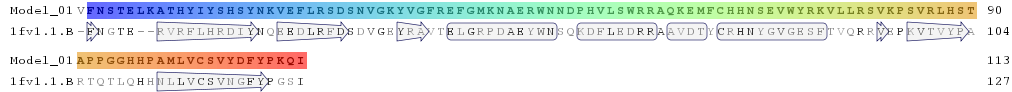


Amci-DXB*0009


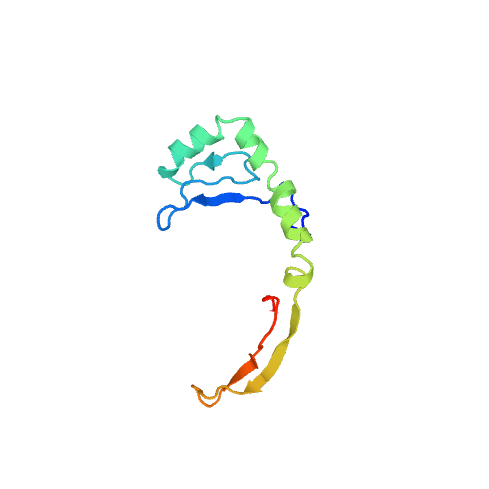

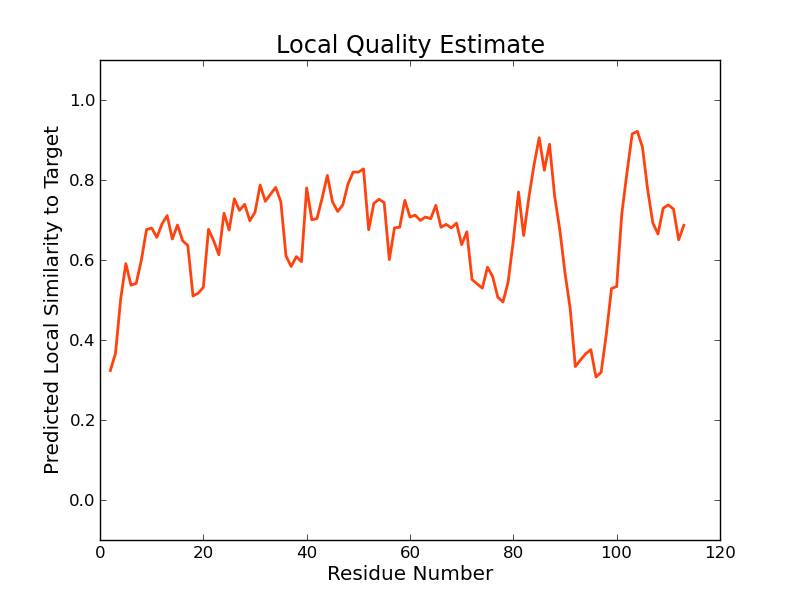

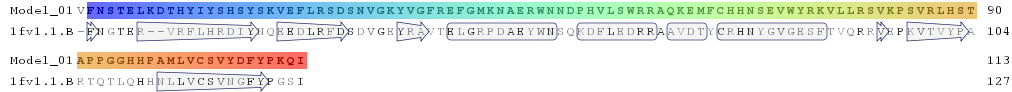


Amci-DXB*0010


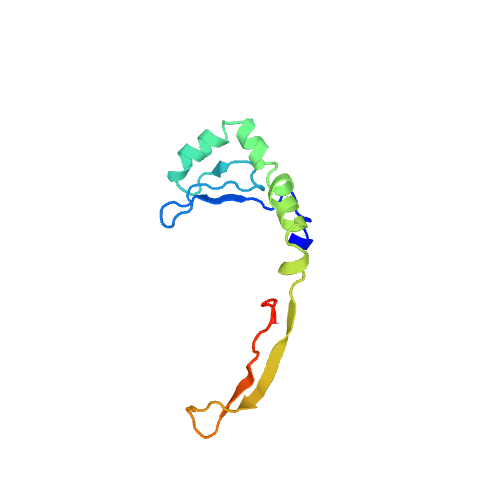

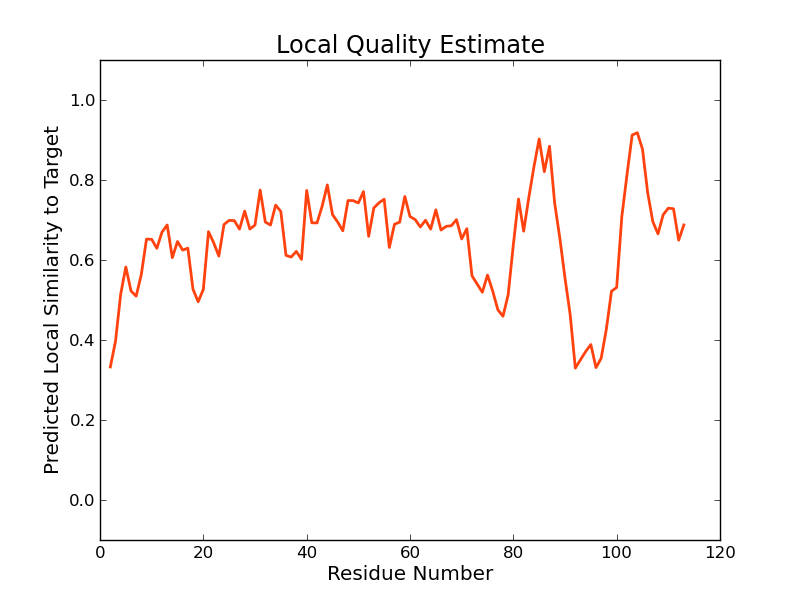

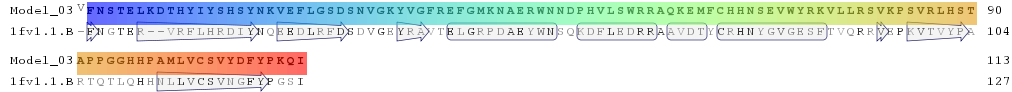


Amci-DXB*0011


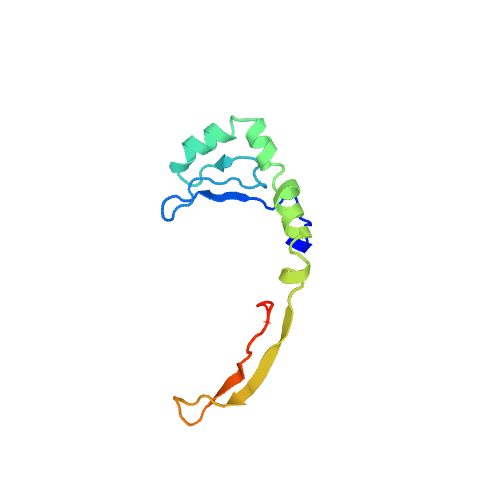

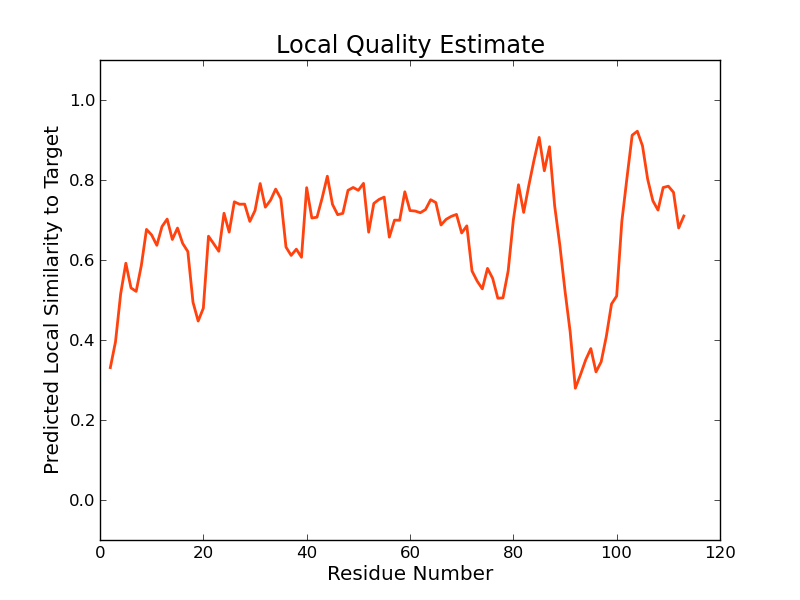

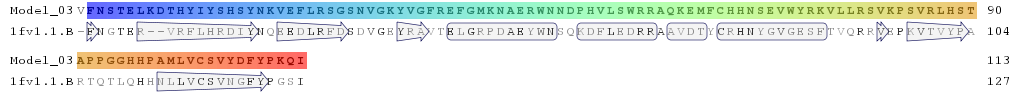


Amci-DXB*0012


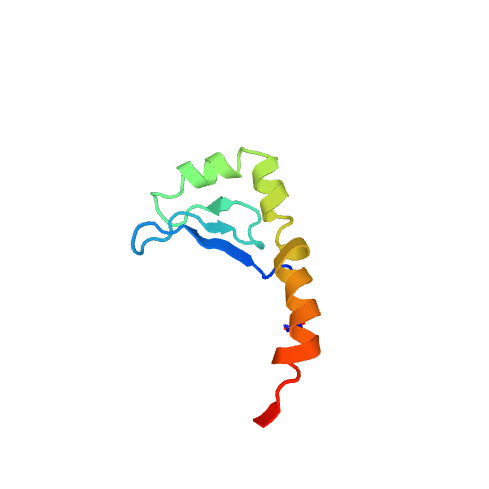

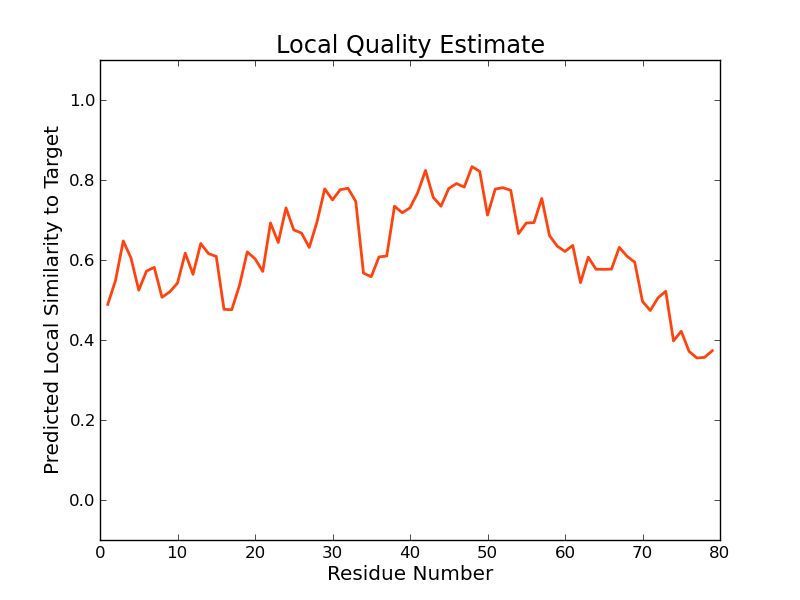


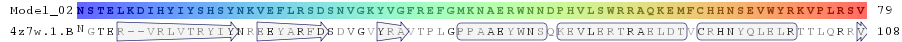


Amci-DXB*0013


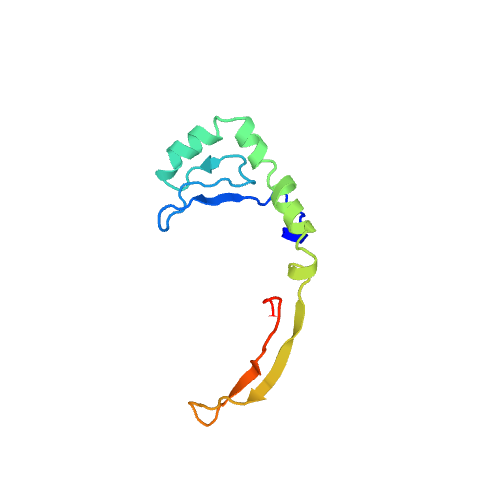

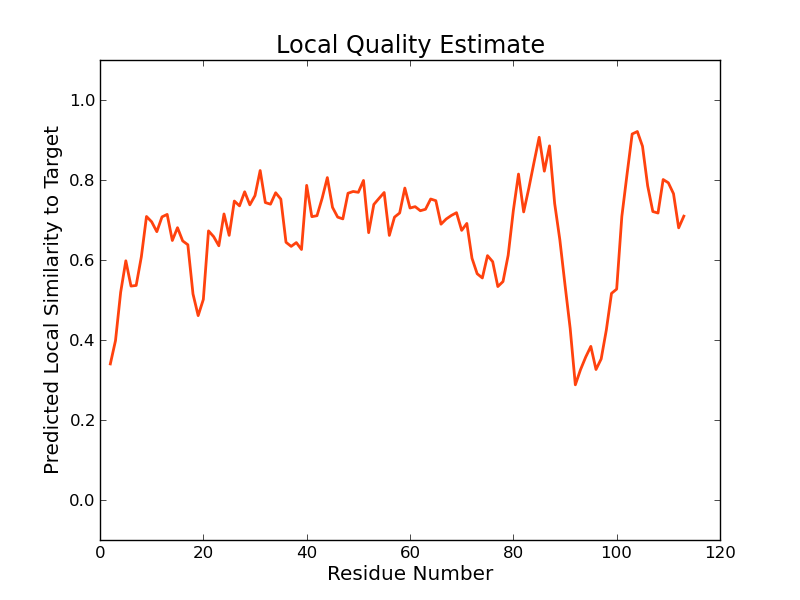

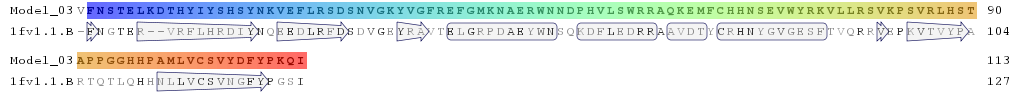


Amci-DXB*0101


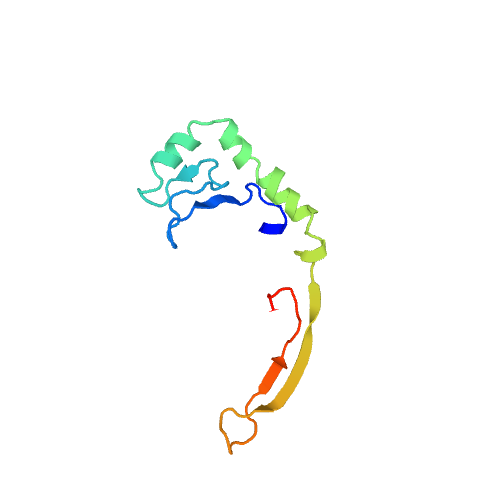

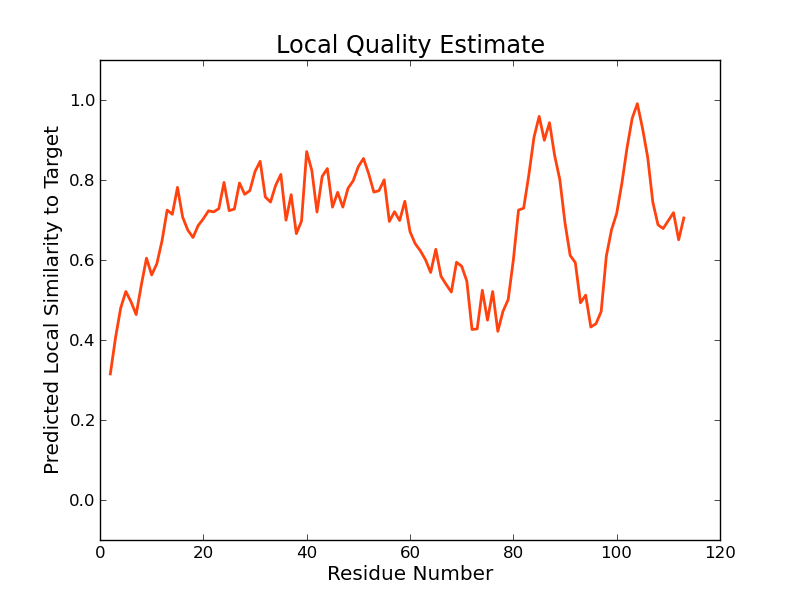

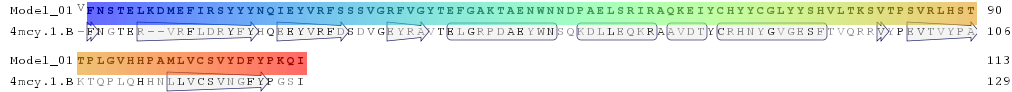


Amci-DXB*0102


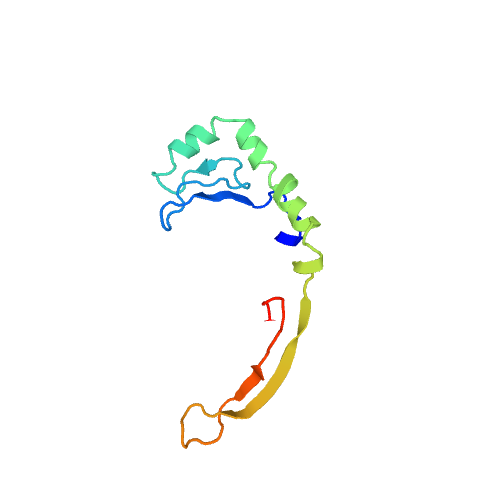

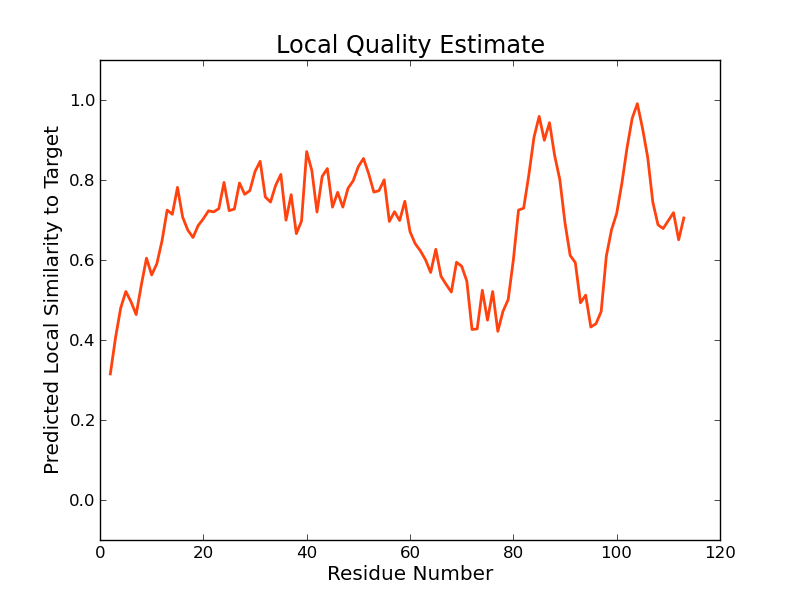

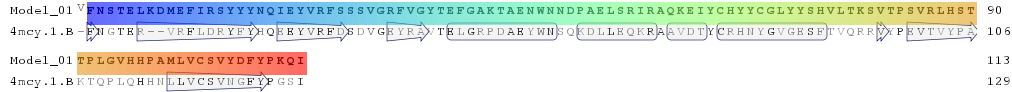


Amci-DXB*0201


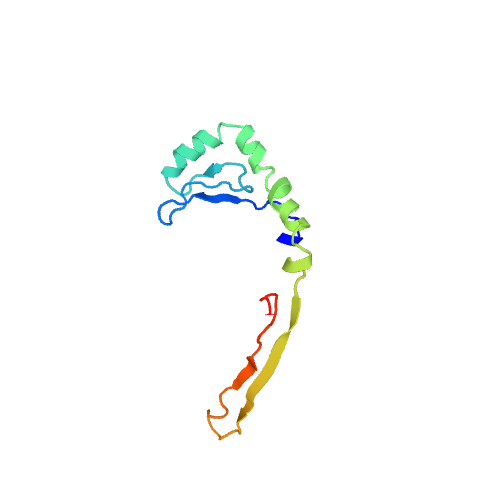

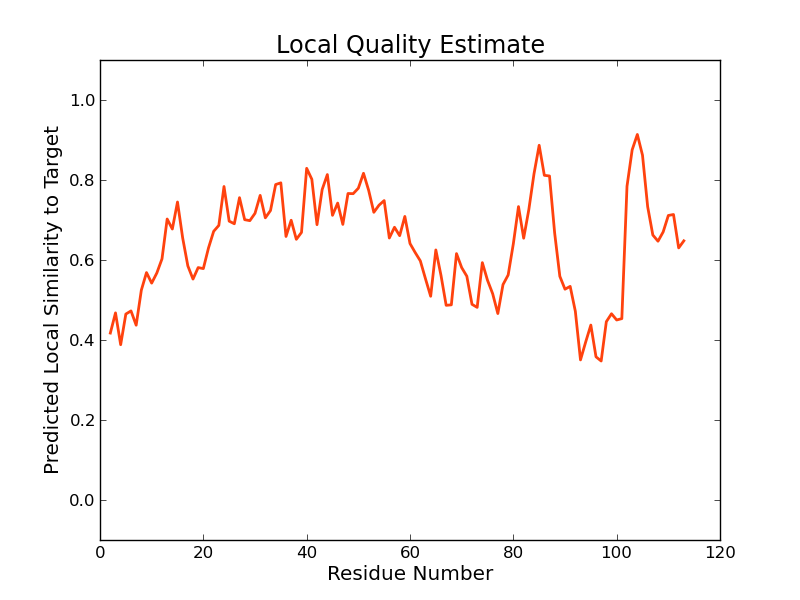

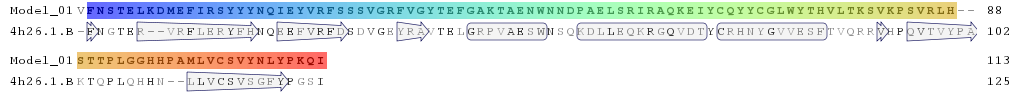


Amci-DXB*0202


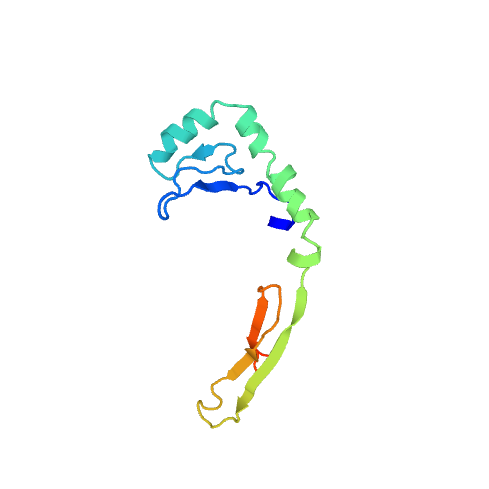

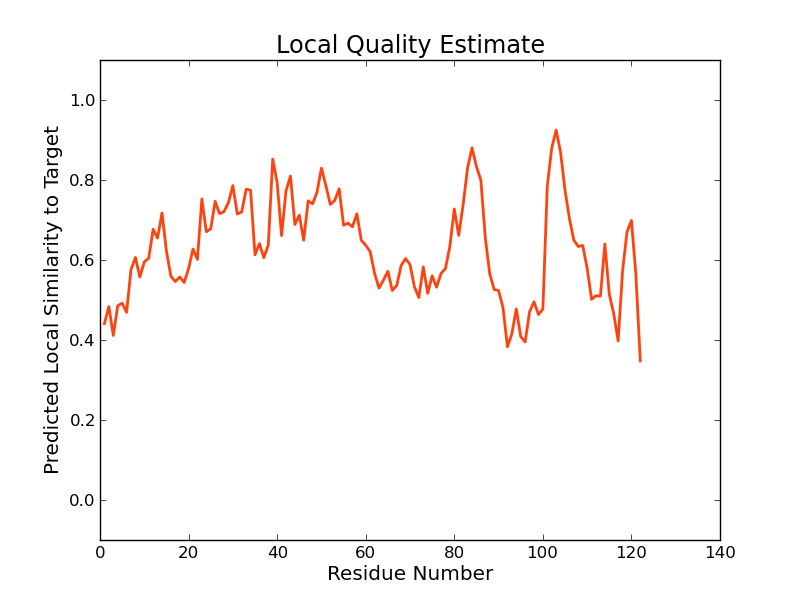


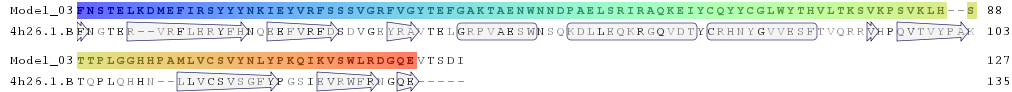


Amci-DXB*0203


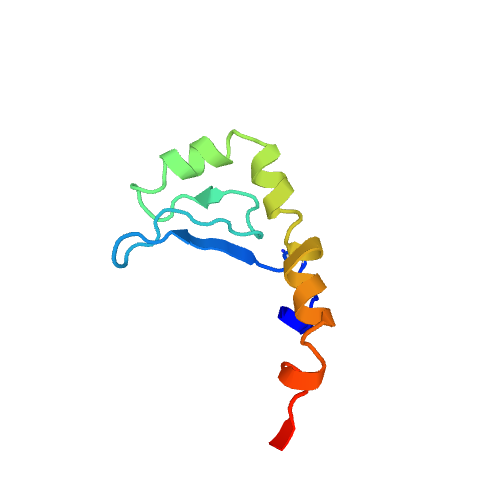

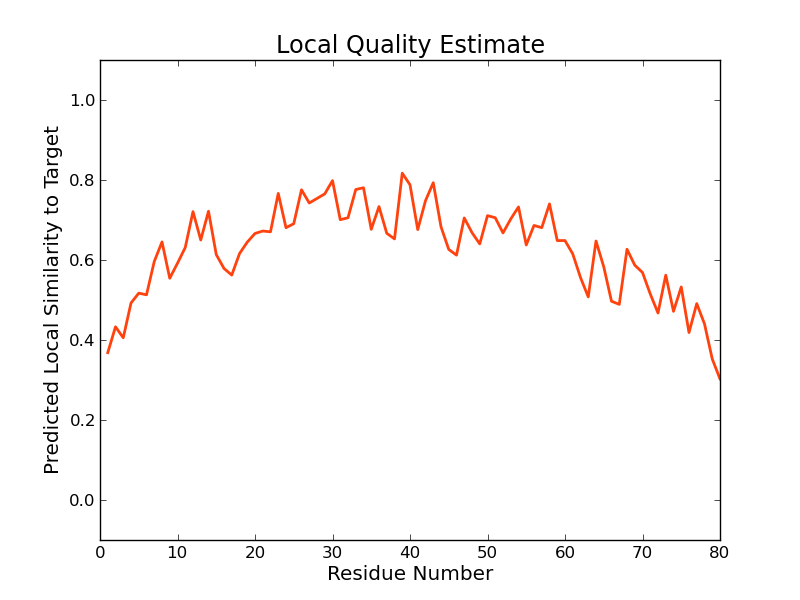

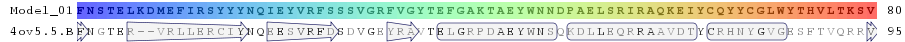


Amci-DXB*03


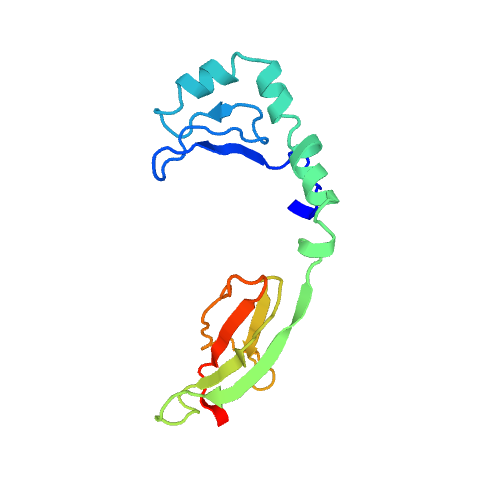

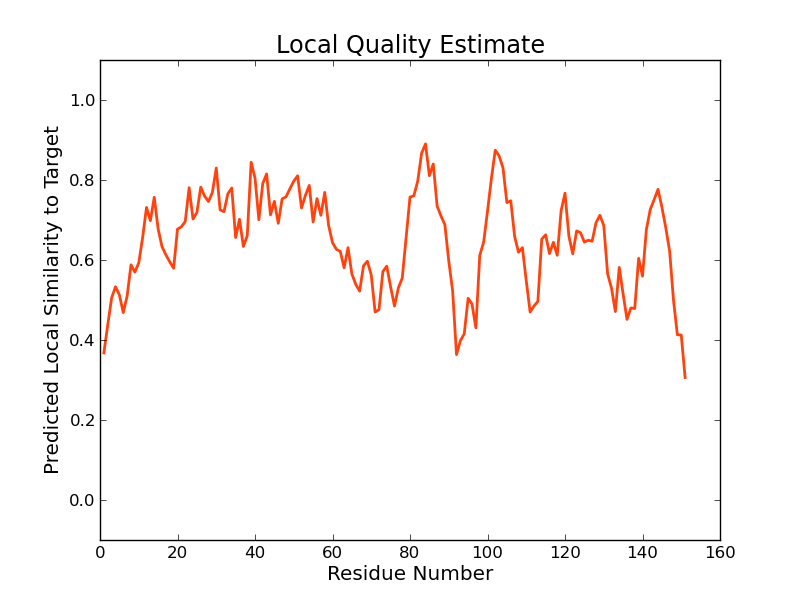

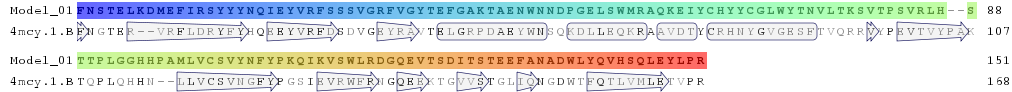


Amci-DXB*040101


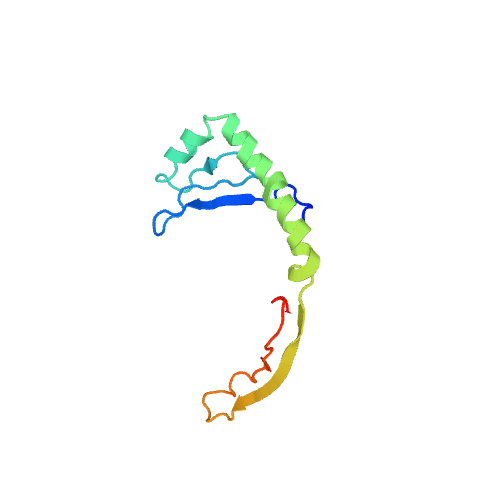

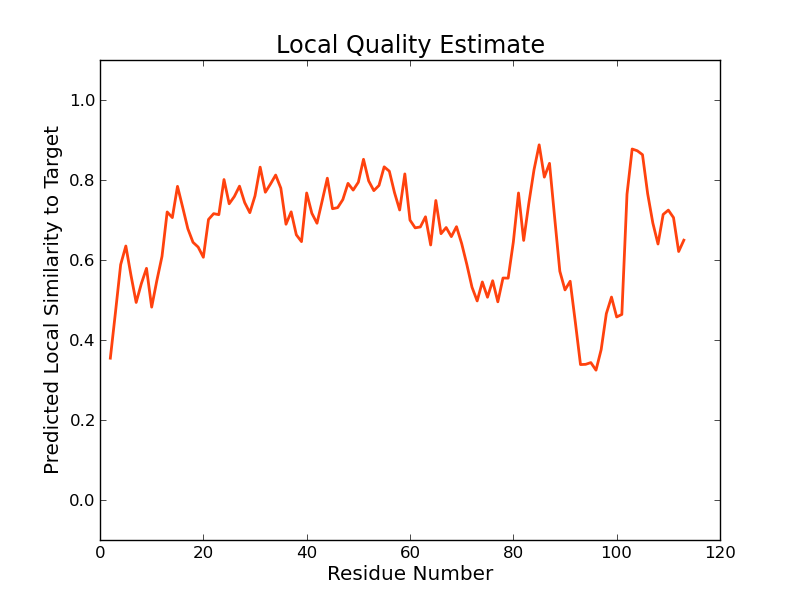

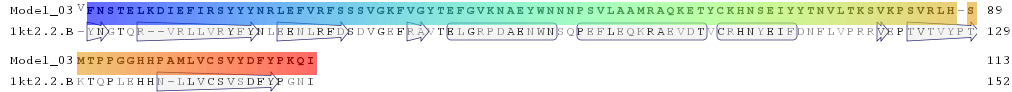


Amci-DXB*040102


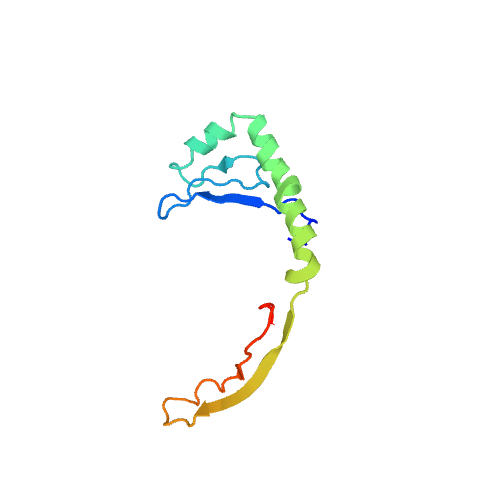

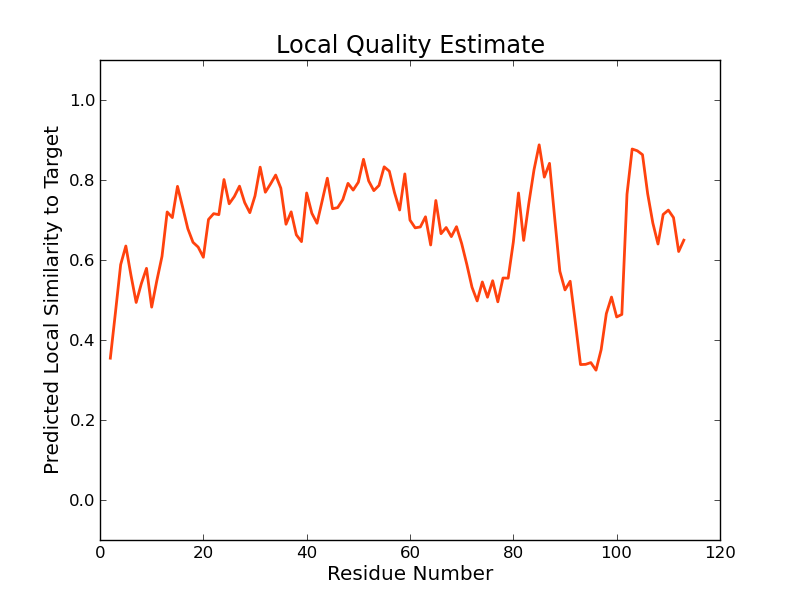

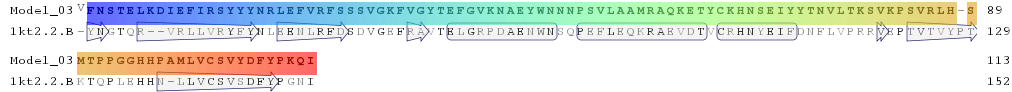


Amci-DXB*040103


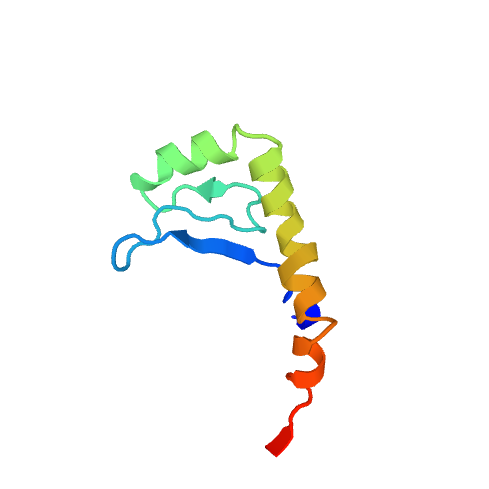

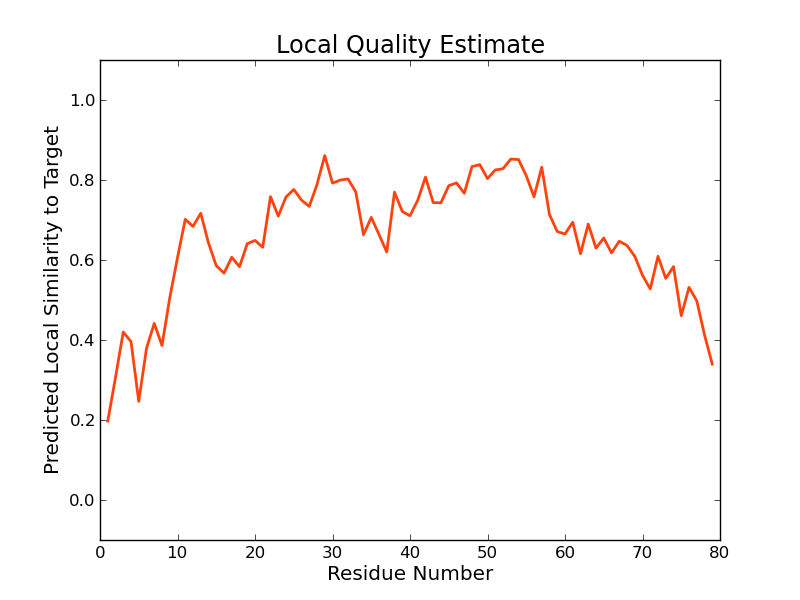

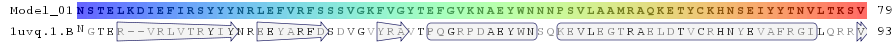


Amci-DXB*0402


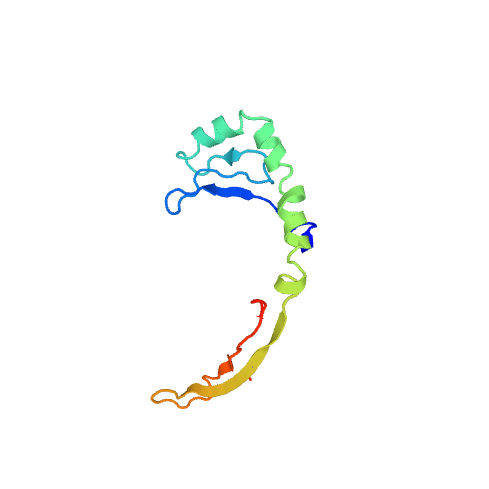

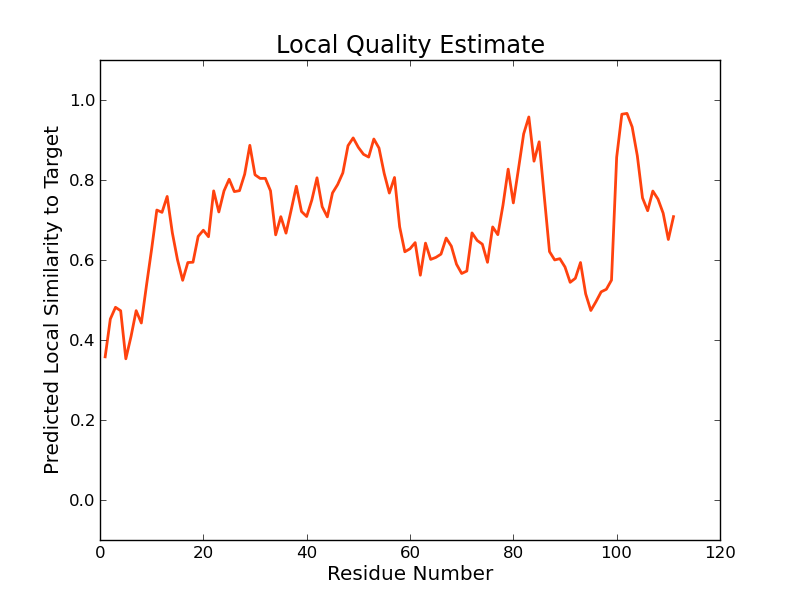

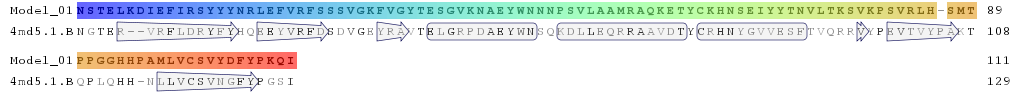


Amci-DXB*040301


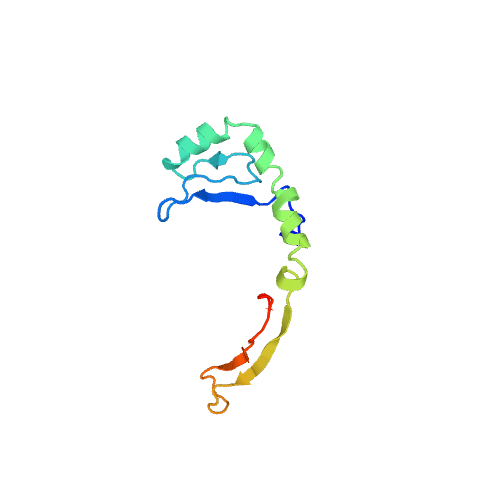

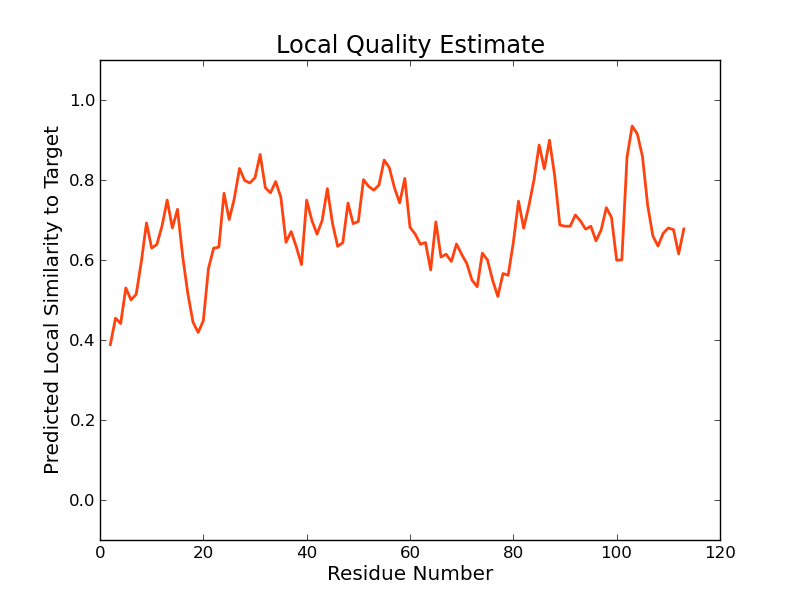

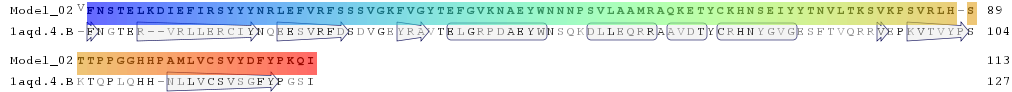


Amci-DXB*040302


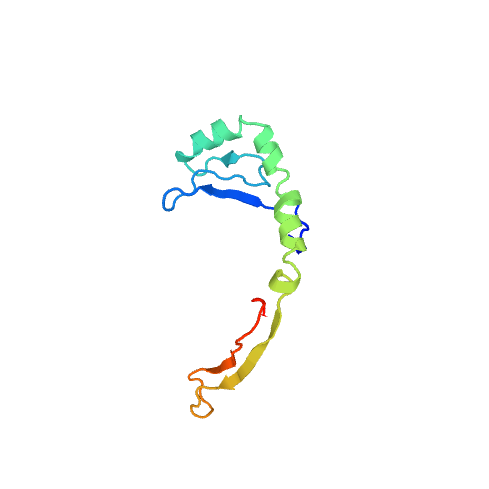

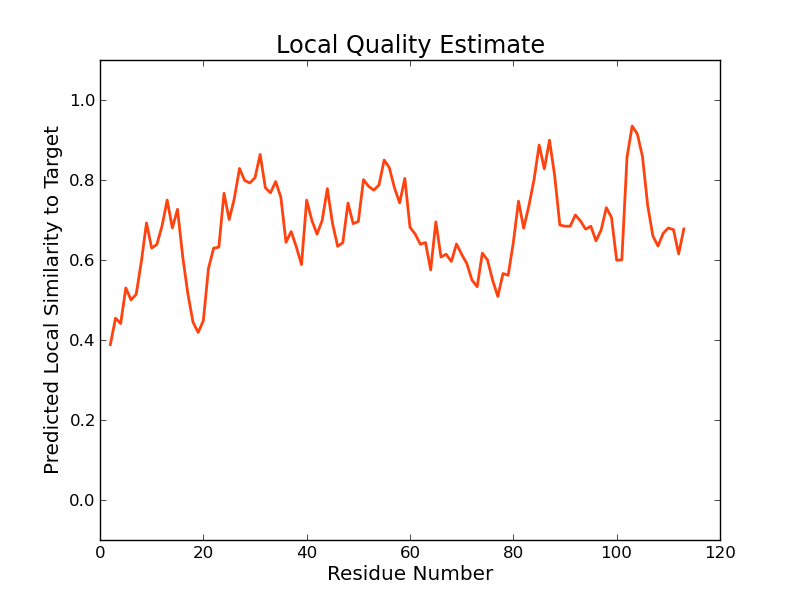

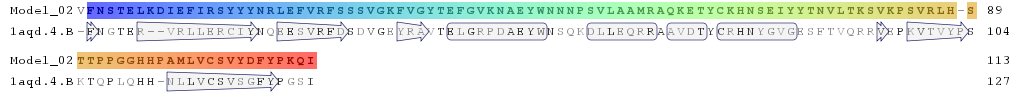


Amci-DXB*040303


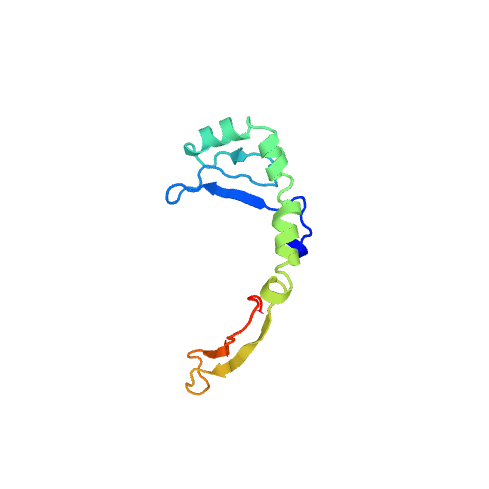

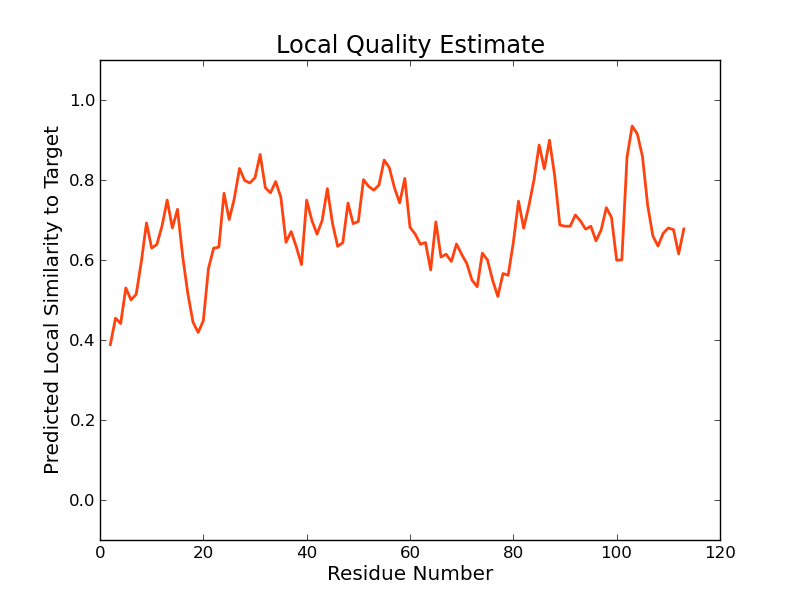


Amci-DXB*040304

Amci-DXB*040305

Amci-DXB*0404

Amci-DXB*0405

Amci-DXB*0406

Amci-DXB*05

Amci-DXB*060101

Amci-DXB*060102

Amci-DXB*0602

Amci-DXB*0603

Amci-DXB*0604

Amci-DXB*07

Amci-DXB*08

Amci-DXB*09

Amci-DXB*1001

Amci-DXB*1002

Amci-DXB*110101

Amci-DXB*110102

Amci-DXB*1102

Amci-DXB*12

Amci-DXB*13

Amci-DXB*14

Amci-DXB*15

Amci-DXB*16

Amci-DXB*17

Amci-DXB*18

Amci-DXB*19

Amci-DXB*20

Amci-DXB*21

Amci-DXB*2201

Amci-DXB*2202

Amci-DXB*23

Amci-DXB*2401

Amci-DXB*25

Amci-DXB*26

Amci-DXB*27

**Summary of all estimated Z-scores for above 3D models**
